# Genetic landscape of hereditary spastic paraplegias in the Korean population

**DOI:** 10.1038/s41598-026-53107-0

**Published:** 2026-05-30

**Authors:** Mi-Ae Jang, Ja-Hyun Jang, Byoung Joon Kim, Duk Hyun Sung

**Affiliations:** 1 Department of Laboratory Medicine and Genetics, Samsung Medical Center, Sungkyunkwan University School of Medicine, Seoul, Republic of Korea; 2 Department of Neurology, Neuroscience Center, Samsung Medical Center, Sungkyunkwan University School of Medicine, Seoul, Republic of Korea; 3 Department of Physical and Rehabilitation Medicine, Samsung Medical Center, Sungkyunkwan University School of Medicine, Seoul, Republic of Korea

**Keywords:** Spastic paraplegia, hereditary, Genetic profile, High-throughput nucleotide sequencing, Inheritance patterns, Diseases, Genetics

## Abstract

Hereditary spastic paraplegia (HSP) is a genetically neurodegenerative disorder with limited epidemiological data in Korea. This study aimed to characterize the genetic landscape of HSP in a large Korean cohort. We analyzed 657 patients with suspected HSP using Sanger sequencing and a targeted next-generation sequencing (NGS) panel covering 54 HSP-related genes. Variants were classified according to American College of Medical Genetics and Genomics/Association for Molecular Pathology guidelines, and copy number variations (CNVs) were detected using in-house methods and multiplex ligation-dependent probe amplification. Pathogenic or likely pathogenic variants were identified in 121 patients (18%), with *SPAST* (83%) and *ATL1* (6%) being the most frequently mutated genes. Notably, 5% of *SPAST* mutations were CNVs, underscoring the importance of CNV detection. Among the identified variants, 23 novel mutations were discovered, primarily in *SPAST* (19), *REEP1* (3), and *SACS* (1), expanding the mutational spectrum of HSP. The diagnostic yield was higher with NGS (25%) compared to Sanger sequencing (16%), reflecting the panel’s broader gene coverage. This study highlights the predominance of *SPAST* and *ATL1* mutations in Korean HSP patients and emphasizes the utility of targeted NGS panels, particularly for detecting CNVs and novel variants. Despite these advances, the majority of cases remain unresolved, suggesting the need for broader sequencing approaches to uncover additional genetic factors. These findings provide a foundation for improved diagnostic strategies and personalized treatment in HSP.

**Supplementary Information:**

The online version contains supplementary material available at 10.1038/s41598-026-53107-0.

## Introduction

Hereditary spastic paraplegia (HSP) represents a diverse group of rare inherited neurodegenerative disorders characterized by progressive weakness and spasticity of both lower extremities^1^. The estimated prevalence of HSP ranges from 1 to10 per 100,000 individuals, with notable regional variation^2^. In European populations, prevalence rates are relatively high (e.g., Spain: 9.6, Norway: 7.4 per 100,000), while in East Asian populations, including Japan, rates are significantly lower (0.1 to 0.9 per 100,000)^2^. Epidemiological data specific to Korea remain limited. According to nationwide, population-based statistics from the Health Insurance Review & Assessment Service (https://opendata.hira.or.kr/or/orb/bigInfo.do, last accessed on May 2025), the number of diagnosed HSP cases in Korea increased five-fold from 168 in 2010 to 861 in 2023. Concurrently, healthcare reimbursement costs for HSP patients rose approximately eight-fold, from 188,154 KRW in 2010 to 1,499,869 KRW in 2023, reflecting the increasing prevalence and growing economic burden on both patients and society.

HSP is one of the most genetically and clinically heterogeneous neurological disorders^1^. Historically, genetic loci associated with HSP were designated SPG (for “spastic paraplegia”) and numbered sequentially based on the order of their discovery. To date, over 80 SPG loci have been identified, with various modes of inheritance reported, including autosomal dominant (AD), autosomal recessive (AR), X-linked (XL), and mitochondrial inheritance^1,3,4^. Clinically, HSP is classified into ‘pure’ and ‘complicated’ types based on the presence of additional neurological or extra-neurological features^1^. The extensive genetic and phenotypic diversity, coupled with phenotypic overlap with other neurological disorders such as adrenoleukodystrophy (ALD)^5,6^, poses significant challenges for accurate genetic diagnosis in clinical practice.

Sanger sequencing targeting high-frequency genes, such as *SPAST* (SPG4) or *ATL1* (SPG3A), served as the primary method for genetic diagnosis^7^. However, with the advent of next-generation sequencing (NGS), multigene panels and exome sequencing have enabled high-throughput analysis of the growing number of genes associated with HSP^8,9^. Owing to practical limitations related to data processing and interpretation, clinical laboratories often prioritize targeted gene panels that focus on well-established HSP-associated genes. This approach streamlines analysis and ensures efficient identification of pathogenic variants (PVs) of Likely pathogenic variants (LPVs) in genes most relevant to the patient’s phenotype while adhering to ethical guidelines^10,11^. In this study, we employed a targeted NGS panel to analyze the clinical and genetic profiles of 657 patients with suspected HSP who underwent genetic testing at our center between 2010 and 2024. Our analysis encompassed inheritance patterns, phenotypic subtypes, the mutational spectrum, and the prevalence of PV or LPV in HSP-related genes.

## Results

### Patient characteristics

We enrolled 657 unrelated patients with suspected HSP in this study, 449 males and 208 females. Table 1 summarizes the demographic data of the genetically solved and unsolved individuals categorized by sex and age groups. The median age of patients at the time of genetic testing was 49 years (range, 1–82), and there was no age difference between the genetically solved group and the unsolved group (Table 1). The age distribution of the study cohort at the time of genetic testing with HSP is as follows: 65 patients aged 10–19 years (10%), 76 patients aged 20–29 years (12%), 76 patients aged 30–39 years (12%), 112 patients aged 40–49 years (17%), 158 patients aged 50–59 years (24%), 119 patients aged 60–69 years (18%), and 51 patients aged over 70 years (8%). Two-thirds of the study population were in the over-40 age group, with the largest number in the 50–59 age group.

**Table 1:** Demographic characteristics of genetically solved and unsolved patients with suspected hereditary spastic paraplegia by sex and age group.

Characteristic	Total (*N* = 657)	Solved (*N* = 121)	Unsolved (*N* = 536)	*P* ^*^
Sex				
Male	449 (68)	71 (59)	378 (71)	0.013
Female	208 (32)	50 (41)	158 (29)	
Age, median (range)	49 (1–82)	48 (1–79)	50 (1–82)	0.447
Number of patients by age at genetic testing				
0–19 years	65 (10)	8 (7)	57 (11)	0.037
20–29 years	76 (12)	12 (10)	64 (12)	
30–39 years	76 (12)	16 (13)	60 (11)	
40–49 years	112 (17)	29 (24)	83 (15)	
50–59 years	158 (24)	34 (28)	124 (23)	
60–69 years	119 (18)	18 (15)	101 (19)	
70–82 years	51 (8)	4 (3)	47 (9)	

Values are presented as number (%) for categorical variables.

^*^The *P* value was obtained by comparing the solved and unsolved groups.

### Frequency and gene spectrum of genetically solved cases

Among the 657 unrelated patients with suspected HSP, 121 (18%) were classified as genetically solved (Figure S1): 114 patients (17.3% of the cohort; 94% of solved cases) carried variants in established HSP-associated genes, including *SPAST* and *ATL1*, while seven patients (1.1% of the cohort; 6% of solved cases) had variants in non-classical HSP-related genes (*ABCD1*, *CYP27A1*, *SACS*). The clinical findings and genetic results of these patients are detailed in Table S1. When comparing the solved percentages by age group, the highest rate (26%) was observed in the 40–49 age group, exhibiting a bell-shaped curve as age increased or decreased from that group, reaching lows of 8% in the over-70 age group and 12% in the 0–19 age group (Figure S2A). The most frequently mutated gene was *SPAST* (affecting 101 individuals, 83% of all solved cases), followed by *ATL1* (7 individuals, 6%), highlighting these genes as major contributors to HSP in this study cohort (Figure S2B). The remaining 6 mutated genes (*REEP1*,* ABCD1*, *KIF5A*,* ALDH18A1*,* SACS*, and *CYP27A1*) were involved in 13 individuals (11%). Mutated genes were mainly associated with AD transmission (*SPAST*, *ATL1*, *REEP1*,* KIF5A*, and *ALDH18A1*; 114 individuals, 94%), followed by XL transmission (*ABCD1*; 5 individuals, 4%), and AR transmission (*SACS* and *CYP27A1*; 2 individuals, 2%) (Figure S2C).

### Mutational analysis of *SPAST*

**Fig. 1:**
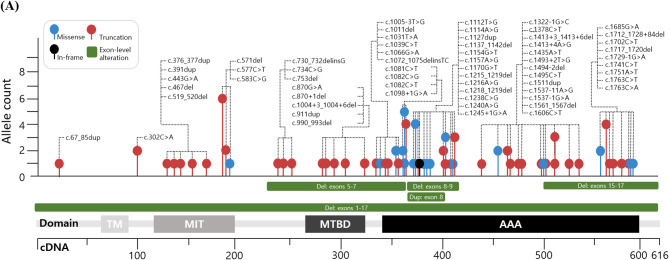

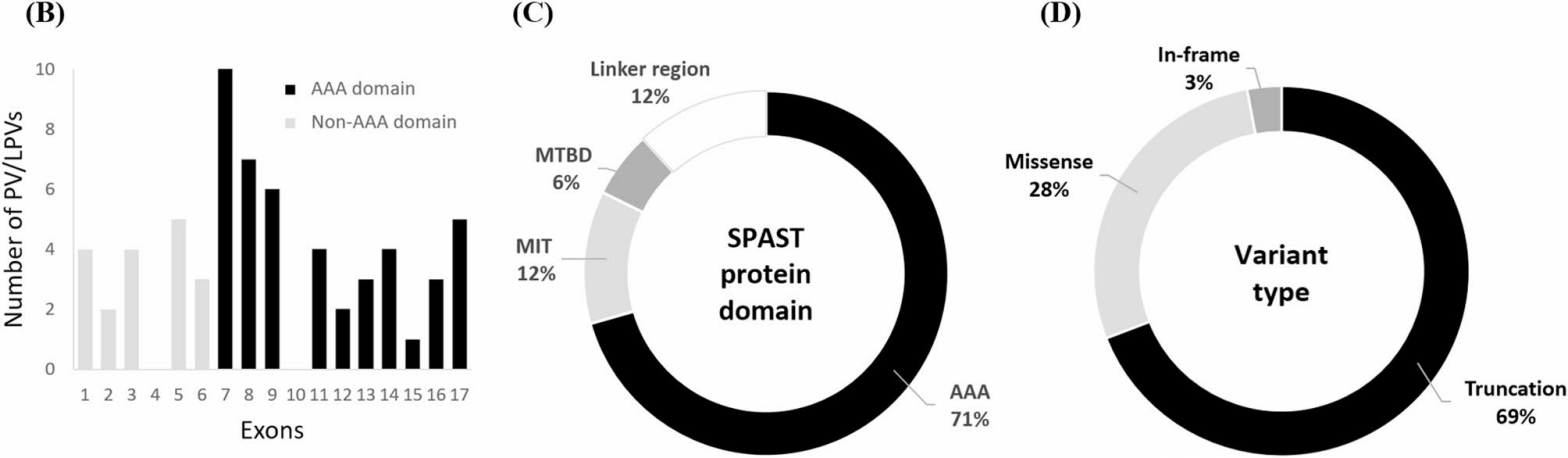
Pathogenic or likely pathogenic variants identified in *SPAST*. (**A**) A schematic representation of the *SPAST* transcript (NM_014946.3) showing the position and frequency of the variants identified in this study. The symbol color indicates the variant type, and protein domains are depicted below the transcript. (**B**) Distribution of variants within 17 exons of *SPAST*. (**C**) Percentage of sequence variants according to SPAST protein domains. (**D**) Proportion of each variant type. AAA, ATPases associated with diverse cellular activities ; Del, deletion; LPV, likely pathogenic variant; MIT, microtubule interacting and trafficking; MTBD, microtubule-binding domain; PV, pathogenic variant; SNV, single nucleotide variant; TM, transmembrane.

In our study, *SPAST* (SPG4) was the most frequently mutated gene; it was identified in 101 patients (15% of the total cohort). The majority of patients (95%, 96/101) harbored PV or LPV as small nucleotide changes including substitutions (62 individuals, 61%), deletions (26, 26%), duplications (6, 6%), and deletion-insertions (2, 2%). A minority of patients (5%, 5/101) exhibited exon-level alterations, such as deletions (4, 4%) and duplications (1, 1%) (Fig. 1A). In total, 68 distinct PV or LPV in *SPAST* were identified, of which 49 have been reported or listed in the Human Gene Mutation Database (HGMD) professional (v2025.1) or ClinVar (https://www.ncbi.nlm.nih.gov/clinvar, last accessed July 2025); the remaining 19 variants are novel. The novel variants were detailed in Table 2.

**Table 2:** Novel pathogenic or likely pathogenic variants identified in 121 genetically solved patients and their characteristics.

Gene	Exon	Nucleotide change	Predicted effect	Current study		Zygosity	In-silico prediction	Class	ACMG criteria
				Allele count	AF % (95% CI)				
*SPAST*	Exon 1	c.391dup	p.(Leu131Profs*5)	1	0.4 (0.1–2.3)	Het	Predicted to undergo NMD	LPV	PVS1, PM2
	Exon 2	c.467del	p.(Leu156Argfs*5)	1	0.4 (0.1–2.3)	Het	Predicted to undergo NMD	LPV	PVS1, PM2
	Exon 5	c.730_732delinsG	p.(Asn244Valfs*21)	1	0.4 (0.1–2.3)	Het	Predicted to undergo NMD	LPV	PVS1, PM2
	Intron 7	c.1005-3T > G	p.?	1	0.4 (0.1–2.3)	Het	SpliceAI (0.98 for acceptor loss; 0.87 for acceptor gain)	LPV	PVS1 (RNA), PM2
	Exon 7	c.1011del	p.(Ala338Leufs*7)	1	0.4 (0.1–2.3)	Het	Predicted to undergo NMD	LPV	PVS1, PM2
	Exon 7	c.1072_1075delinsTC	p.(Val358Serfs*8)	1	0.4 (0.1–2.3)	Het	Predicted to undergo NMD	LPV	PVS1, PM2
	Exon 8	c.1127dup	p.(Gly377Argfs*17)	1	0.4 (0.1–2.3)	Het	Predicted to undergo NMD	LPV	PVS1, PM2
	Exon 8	c.1154G > T	p.(Gly385Val)	1	0.4 (0.1–2.3)	Het	REVEL (0.978)	LPV	PM5, PM1, PM2, PP2, PP3
	Exon 8	c.1170G > T	p.(Met390Ile)	1	0.4 (0.1–2.3)	Het	REVEL (0.900)	PV	PS1, PM5, PM2, PP2, PP3
	Exon 8	c.(1098+1_1099-1)_ (1173+1_1174-1)dup	-	1	0.4 (0.1–2.3)	Het	Not predicted to undergo NMD	LPV	PVS1_S, PM1, PM2
	Exon 9	c.1218_1219del	p.(Ile406Metfs*36)	1	0.4 (0.1–2.3)	Het	Predicted to undergo NMD	LPV	PVS1, PM2
	Exon 9	c.1240 A > G	p.(Lys414Glu)	2	0.8 (0.2–3.0)	Het	REVEL (0.974)	LPV	PM1, PM5_P, PM2, PP2, PP3
	Intron 10	c.1322-1G > C	p.?	1	0.4 (0.1–2.3)	Het	SpliceAI (0.98 for acceptor loss)	PV	PVS1, PM2, PS1_P
	Exon 12	c.1435 A > T	p.(Arg479*)	1	0.4 (0.1–2.3)	Het	Predicted to undergo NMD	LPV	PVS1, PM2
	Intron 12	c.1493 + 2T > G	p.?	1	0.4 (0.1–2.3)	Het	SpliceAI (1.00 for donor loss)	PV	PVS1, PM2, PS1_P
	Exon 14	c.1561_1567del	p.(Leu521Valfs*7)	1	0.4 (0.1–2.3)	Het	Predicted to undergo NMD	LPV	PVS1, PM2
	Exon 16	c.1712_1728 + 84del	p.(Glu563Aspfs*8)	4	1.7 (0.6–4.2)	Het	SpliceAI (0.98 for donor loss)	LPV	PVS1_S (RNA), PS4_M, PM2
	Exon 16	c.1717_1720del	p.(Ser573Profs*4)	1	0.4 (0.1–2.3)	Het	Not predicted to undergo NMD	LPV	PVS1_S, PM2
	Exon 17	c.1763 C > T	p.(Ser588Phe)	1	0.4 (0.1–2.3)	Het	REVEL (0.909)	LPV	PM5, PM1, PM2, PP3
*REEP1*	Exon 4	c.302del	p.(Lys101Argfs*26)	1	0.4 (0.1–2.3)	Het	Predicted to undergo NMD	LPV	PVS1, PM2
	Intron 4	c.303 + 1G > T	p.?	1	0.4 (0.1–2.3)	Het	SpliceAI (1.00 for donor loss)	PV	PVS1, PM2, PS1_P
	Exons 6–7	c.(417+1_418-1)_ (*3107_? )del	-	1	0.4 (0.1–2.3)	Het	Predicted to undergo NMD	LPV	PVS1, PM2
*SACS*	Exon 10	c.13217del	p.(Thr4406Argfs*46)	1	0.4 (0.1–2.3)	Het	Not predicted to undergo NMD	LPV	PVS1_S, PM2, PM3

The reference transcripts used for *SPAST*, *REEP1*, and *SACS* were NM_014946.3, NM_022912.2, and NM_014363.5, respectively.

ACMG, American College of Medical Genetics and Genomics; AF, allele frequency; CI, confidence interval; Het, heterozygous; LPV, likely pathogenic variant; PV, pathogenic variant; NMD, nonsense-mediated decay.

**Fig. 2:**
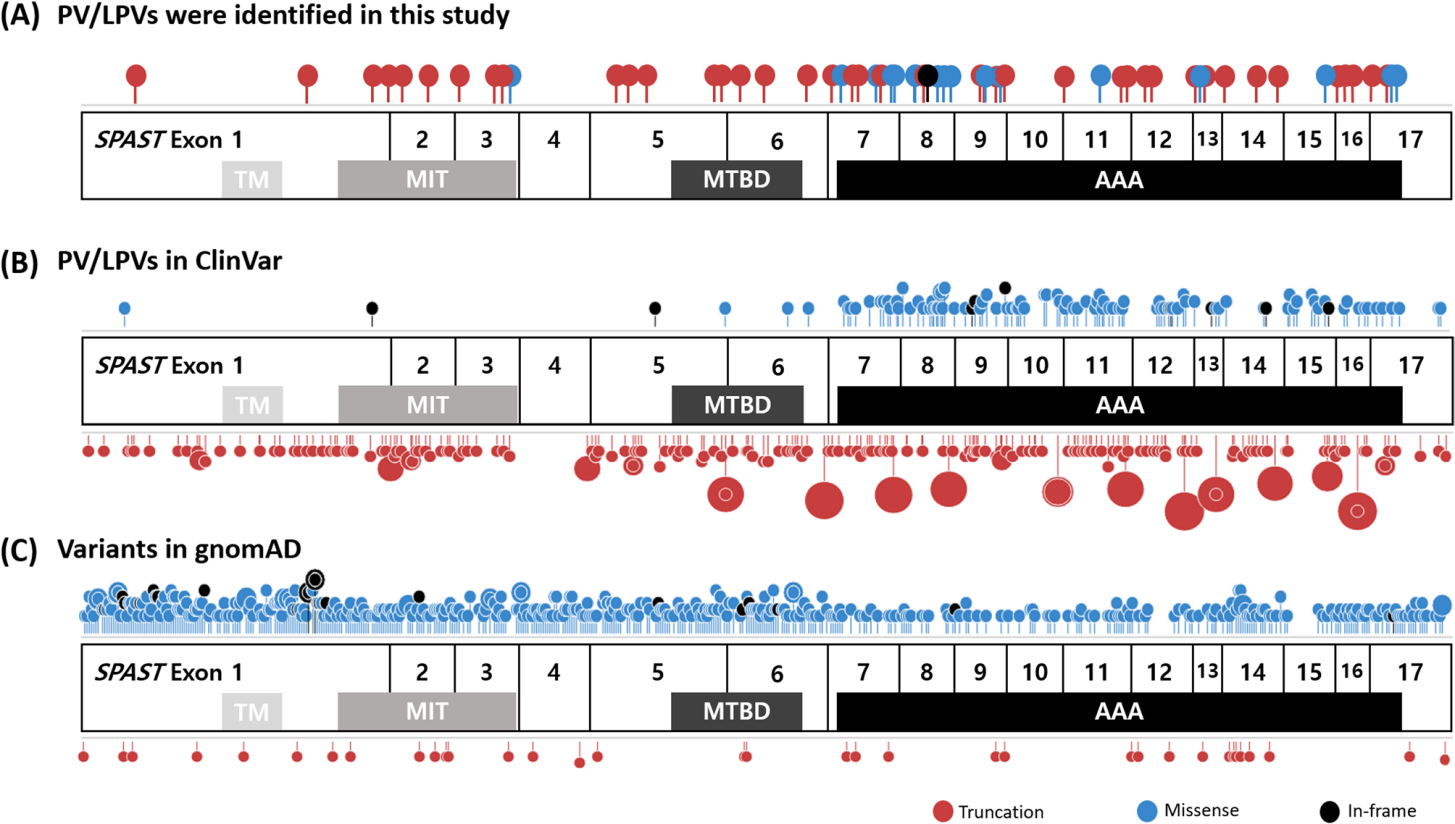
Comparison of frequency and location of *SPAST* variants from the (**A**) current study, (**B**) ClinVar (https://www.ncbi.nlm.nih.gov/clinvar/), and (**C**) gnomAD database (https://gnomad.broadinstitute.org/). The symbol color indicates the variant type. Exon-level deletion/duplication variants are not shown here. AAA, ATPases associated with diverse cellular activities; LPV, likely pathogenic variant; MIT, microtubule interacting and trafficking; MTBD, microtubule-binding domain; PV, pathogenic variant; TM, transmembrane.

All identified PV or LPV were mapped along the entire SPAST protein (Fig. 1A). The variants were widely distributed across *SPAST* exons, predominantly located within the ATPase Associated with various cellular Activities (AAA) cassette domain (residues 342–599), which accounted for 71% (48/68) of small nucleotide changes (Fig. 1B and C). Most *SPAST* PV or LPV were truncation (47 variants, 69%), including frameshift, splice site, nonsense, and exon-level deletion/duplication variants; the remaining were missense (19, 28%) and in-frame deletion/duplication (2, 3%) variants (Fig. 1D). In our cohort, truncation variants were found throughout SPAST protein, whereas missense PV or LPV were notably enriched within the AAA cassette domain (Fig. 2A). This observation was corroborated by analysis of the ClinVar database, which also showed a similar pattern of missense PV or LPV concentrated in the AAA domain (Fig. 2B). Conversely, data from the gnomAD database (v4.1.0) indicated that missense and in-frame variants were significantly enriched outside of the AAA domain (Fig. 2C). The AAA cassette domain, a highly conserved region of approximately 250 amino acids, is essential for ATP binding and hydrolysis^12^. This energy-dependent mechanism is vital for Spastin’s role in severing microtubules, a process crucial for axonal transport and neuronal morphology^13,14^. Missense variants within this region would predictably disrupt the proper folding, oligomerization, or ATP-hydrolyzing activity of Spastin, potentially leading to impaired microtubule dynamics and the HSP phenotype.

### Validation of candidate *SPAST* variants via RT-PCR and sequencing

We performed validation using RT-PCR and sequencing for three candidate *SPAST* variants, c.1004+3_1004 + 6del, c.1005-3T > G, and c.1712_1728 + 84del, which were predicted to affect splicing, but this was not previously experimentally confirmed (Figure S3). The c.1004+3_1004 + 6del variant resulted in exon 6 skipping (r.871_1004del), which led to frameshift and premature stop codon, p.(Gly291Valfs*10) (Figure S3A). RNA study revealed that the c.1005-3T > G variant activated a cryptic acceptor site at upstream 22 bp region, which led to a 25-bp insertion between exon 6 and exon 7 (r.1004_1005ins[1005-25_1005-4;gag]); this was predicted to create premature stop codon p.(Asn335Lysfs*4) (Figure S3B). The c.1712_1728 + 84del variant corresponded to a 101-bp deletion resulting in skipping of exon 16 (r.1688_1728del), which was predicted to cause a frameshift and creation of premature stop codon p.(Glu563Aspfs*8) (Figures S3C and S3D).

### Validation of multi-exon deletions in *SPAST* using MLPA

Of 142 patients who underwent panel-based NGS testing, five were suspected to have CNVs involving *SPAST.* These CNVs included deletions in exons 5–7, 8–9, 15–17, and a whole gene deletion covering exons 1–17, as well as a duplication in exon 8. All detected CNVs were present in a heterozygous state. Subsequent MLPA analysis confirmed that all CNV calls identified by NGS were consistent with the MLPA results (Figure S4).

### Mutational analysis of *ATL1*, *REEP1*, and other rare HSP genes

*ATL1* (SPG3A) was the second most common AD-HSP gene in this study cohort. In our cohort, we identified six PV or LPV, c.35–3 C > T [p.?], c.536 C > A [p.(Ser179Tyr)], c.715 C > G [p.(Arg239Cys)], c.740 A > C [p.(His247Pro)], c.1193 C > T [p.(Ser398Phe)], and c.1483 C > T [p.(Arg495Trp)], of *ATL1* in seven SPG3A patients. There were no functional domain where PV or LPV were clustered, and the c.35–3 C > T [p.?] was the only recurrent variant that occurred in two patients (Table 3).

**Table 3:** Recurrent pathogenic or likely pathogenic variants identified in 121 genetically solved patients and their characteristics.

Gene	Exon	Nucleotide change	Predicted effect	Current study		Protein domain	Variant type	Comment
				Allele count	AF % (95% CI)			
*SPAST*	Exon 3	c.571del	p.(Arg191Alafs*5)	6	2.5 (1.2–5.4)	MIT	Truncation	Predicted to undergo NMD
	Exon 7	c.1082 C > G	p.(Pro361Arg)	5	2.1 (0.9–4.8)	AAA	Missense	Disrupts p.Pro361 residue, which has also been previously altered with Ser or Leu in *SPAST*-related conditions
	Intron 7	c.1098 + 1G > A	p.?	4	1.7 (0.7–4.2)	AAA	Truncation	Exon 7 skipping disrupts reading frame, predicted to undergo NMD
	Exon 8	c.1114 A > G	p.(Arg372Gly)	4	1.7 (0.7–4.2)	AAA	Missense	Disrupts p.Arg372 residue, which has also been previously altered with Ser or Thr in *SPAST*-related conditions
	Exon 16	c.1712_1728 + 84del	p.(Glu563Aspfs*8)	4	1.7 (0.7–4.2)	AAA	Truncation	Validated as r.1688_1728del (exon 16 skipping) by our RNA analysis
	Exon 9	c.1216 A > G	p.(Ile406Val)	3	1.3 (0.4–3.6)	AAA	Missense	Disrupts p.Ile406 residue, which has also been previously altered with Arg in *SPAST*-related conditions
	Intron 9	c.1245 + 1G > A	p.?	3	1.3 (0.4–3.6)	AAA	Truncation	Skipped exon 9, which encodes part of the critical ATPase AAA domain
	Intron 13	c.1537–11 A > G	p.?	3	1.3 (0.4–3.6)	AAA	Truncation	Validated previously as exon 14 and exon 13–14 deletions (PMID, 26208798)
	Exon 1	c.302 C > A	p.(Ser101*)	2	0.8 (0.2–3.1)	-	Truncation	Predicted to undergo NMD
	Exon 3	c.577 C > T	p.(Gln193*)	2	0.8 (0.2–3.1)	MIT	Truncation	Predicted to undergo NMD
	Exon 7	c.1066G > A	p.(Glu356Lys)	2	0.8 (0.2–3.1)	AAA	Missense	Disrupts p.Glu356 residue, which has also been previously altered with Gly in *SPAST*-related conditions
	Exon 7	c.1081 C > T	p.(Pro361Ser)	2	0.8 (0.2–3.1)	AAA	Missense	Disrupts the p.Pro361 amino acid residue
	Exon 9	c.1215_1219del	p.(Asn405Lysfs*36)	2	0.8 (0.2–3.1)	AAA	Truncation	Predicted to undergo NMD
	Exon 9	c.1240 A > G	p.(Lys414Glu)	2	0.8 (0.2–3.1)	AAA	Missense	Disrupts p.Lys414 residue, which has also been previously altered with Thr in *SPAST*-related conditions
	Exon 11	c.1378 C > T	p.(Arg460Cys)	2	0.8 (0.2–3.1)	AAA	Missense	Disrupts p.Arg460 residue, which has also been previously altered with Leu or His in *SPAST*-related conditions
	Intron 11	c.1413+3_1413 + 6del	p.?	2	0.8 (0.2–3.1)	AAA	Truncation	Multiple pathogenic splice site variants within the same splice region exhibiting same predicted impact, such as c.1413 + 3 A > C
	Exon 15	c.1685G > A	p.(Arg562Gln)	2	0.8 (0.2–3.1)	AAA	Missense	Disrupts p.Arg562 residue, which has also been previously altered with Gly in *SPAST*-related conditions
*ATL1*	Intron 1	c.35–3 C > T	p.?	2	0.8 (0.2–3.1)	-	Truncation	Validated previously as exon 2 skipping (PMID 24969372)

The reference transcripts used for *SPAST* and *ATL1* was NM_014946.3 and NM_015915.4, respectively.

AAA, ATPases associated with diverse cellular activities; AF, allele frequency; CI, confidence interval; Het, heterozygous; LoF, loss-of-function; MIT, microtubule interacting and trafficking; NMD, nonsense-mediated decay.

*REEP1* (SPG31) was the third most commonly identified gene associated with AD-HSP in our cohort, following *SPAST* and *ATL1*. Four PV or LPV were identified in *REEP1*, including three small nucleotide changes, c.2T > C [p.Met1?], c.302del [p.(Lys101Argfs*26)], and c.303 + 1G > T [p.?] and a multi-exon deletion involving exons 6 and 7. Each variant was found in a different SPG31 patient, resulting in a total of four patients with *REEP1* PV or LPV (Supplementary Table S1). Of these four variants, three (c.302del, c.303 + 1G > T, and the multi-exon deletion) were novel (Table 2), while the c.2T > C variant has previously been reported in the HGMD and ClinVar (variation ID: 941193).

As rare genetic causes of HSP, we identified PV or LPV in genes of *KIF5A* and *ALDH18A1*. In *KIF5A* (SPG10), a LPV, c.838 C > T [p.(Arg280Cys)], was identified in a male patient (HSP-651) who experienced onset in his early 40s. The patient exhibited progressive weakness and spasticity of both lower extremities without accompanying neurological deficits, suggesting pure HSP type. In *ALDH18A1* (SPG9A), a LPV, c.755G > A [p.(Arg252Gln)], was identified in a 25-year-old female patient (HSP-647) in a heterozygous state. The patient experienced gait disturbances that began four months prior, characterized by frequent falls and difficulty fully extending her knees upon standing. There was no relevant family history. *ALDH18A1* is associated with SPG9A or 9B disorders, which can follow either AD or AR inheritance patterns. The variant identified in this study, c.755G > A [p.(Arg252Gln)], has been previously reported in the literature to be associated with AD inheritance^15^.

### Mutational analysis of HSP-related disorders

Seven patients were identified with PV or LPV associated with HSP-related disorders (Table S2). The most frequently implicated gene was *ABCD1*, linked to ALD. Five patients exhibited PV or LPV in *ABCD1*: four male patients were hemizygous (c.112 C > T, c.250 C > T, c.1876G > A, and c.1895 C > T), and one female patient was heterozygous (c.1628 C > T), representing 0.8% (5/657) of the entire cohort. A male patient with c.112 C > T [p.(Gln38*)] (HSP-453) reported slower walking beginning in his 50s and was clinically diagnosed with cerebellar ataxia before genetic testing. His mother also experienced walking difficulties. The female patient (HSP-482) presented with weakness in the lower legs, more pronounced on the left, with onset in her 50s and no family history. She carried the c.1628 C > T [p.(Pro543Leu)] variant and recalled being unable to run as fast as peers and prone to falls during her youth. Female carriers of *ABCD1* often develop signs of the disease with later onset^16^. Another male patient with the c.1895 C > T [p.(Thr632Ile)] (HSP-048) experienced paralysis in both lower legs when standing for extended periods, and numbness in the right foot began around age 40. His younger brother and maternal uncle exhibited similar symptoms but were not genetically diagnosed. Patients with *ABCD1* PV or LPV showed an elevated C26:0/C22:0 ratio indicating increased serum fatty acid levels (Table S2).

One patient (HSP-504) with cerebrotendinous xanthomatosis and a history of Alzheimer’s disease harbored two PV or LPV in *CYP27A1*, c.1001T > A [p.(Met334Lys)] and c.1420 C > T [p.(Arg474Trp)], presenting with hyperreflexia in her limbs^17^.

Lastly, one patient (HSP-523) had double heterozygous variants in *SACS*, c.2903_2906del [p.(Asp968Valfs*13)) and c.13217del [p.(Thr4406Argfs*46)], causal for Charlevoix-Saguenay-type spastic ataxia. He reported difficulty running and balance problems beginning in his teens. Examination findings included negative plantar response, abnormal Romberg sign, and impaired touch and pain sensation. His older sister harbored the same *SACS* variants. These features such as early gait/balance difficulties, abnormal Romberg, and sensory impairment, illustrate clinical overlap with cerebellar ataxia and related syndromes. The c.13217del is a novel frameshift variant not present in gnomAD (v4.1.0), which was predicted to introduce a premature stop codon, while the opposite allelic variant c.2903_2906del is a known PV, and are together interpreted as a LPV.

## Discussion

Our study represents one of the largest and most comprehensive genetic analyses of HSP in a Korean population, encompassing 657 unrelated patients over a 14-year period. The novelty of this research lies in its extensive cohort size, which provides a robust foundation for understanding the genetic landscape of HSP in Korea. Unlike previous studies that often focused on smaller patient groups or specific genes^7,9,18^, our investigation utilized a broad NGS panel targeting 54 genes associated with HSP and related disorders, allowing for more comprehensive genetic assessment. Despite the genetic heterogeneity of HSP, our findings underscore the predominance of well-known genes, particularly *SPAST* (SPG4) and *ATL1* (SPG3A), which together accounted for nearly 90% of the genetically solved cases. This highlights the importance of these genes in the Korean HSP population and suggests that initial diagnostic strategies should prioritize these genes. In addition, our study revealed that a significant proportion of *SPAST* mutations involved CNVs, emphasizing the need for diagnostic approaches that can detect both small nucleotide changes and larger structural variants. This insight is crucial for improving diagnostic accuracy and ensuring that patients with CNVs are not overlooked in clinical practice.

In our study, PV or LPV were identified in 121 of 657 patients (18%), a diagnostic yield that appears lower compared to studies conducted in other populations^19–22^. This discrepancy may be attributed to several factors, including differences in genetic architecture, methodological approaches employed, and the inclusion of patients with a low likelihood of genetic confirmation despite clinical suspicion of HSP. Our diagnostic workflow used two primary modalities: Sanger sequencing and NGS panel testing, which are routinely applied in clinical laboratory settings. The detection rate was 16% with Sanger sequencing, which primarily targeted *SPAST* and *ATL1*, while the NGS panel, which included 54 genes associated with HSP and related disorders, yielded a higher detection rate of 25% (Figure S1). This improvement can be attributed to the broader gene coverage of the NGS panel, which allowed for the identification of variants in rarer genes such as *KIF5A*, *ALDH18A1*, and *ABCD1*, as well as the detection of structural variants, including CNVs. Notably, 5% of *SPAST* mutations identified in our cohort were CNVs, which would have been missed by Sanger sequencing alone. This highlights the importance of incorporating CNV analysis into diagnostic workflows, particularly for genes like *SPAST*, where structural variants account for a significant proportion of mutations^23^. The relatively low overall diagnostic rate in our study compared to some international cohorts may also reflect the limitations of targeted NGS panels, which, despite their broad coverage, do not encompass the full spectrum of genes implicated in HSP. Recent studies have shown that whole-exome sequencing (WES) can identify PVs in genes like *ETHE1*, *ZFYVE26*, and *RNF170* in cases where panel testing is negative^21,22^. This highlights the need for broader sequencing approaches, such as WES or whole-genome sequencing (WGS), to uncover the genetic causes in unresolved cases. Such strategies could improve diagnostic yield and provide a more comprehensive understanding of HSP’s genetic architecture, ultimately guiding more precise patient management and treatment.

Notably, our study did not identify any definitive cases involving *SPG7* and *SPG11*, despite their inclusion in our NGS panel and their known high mutation frequencies in other populations^8,19,22^. Instead, we identified two inconclusive cases (Table S3). One patient (HSP-259) carried a heterozygous PV in *SPG7*, c.244 C > T [p.(Gln82*)], which has been previously reported in a homozygous state in late-onset HSP^24^. However, the heterozygous presence of this variant in our patient, along with similar symptoms in family members, does not align with the reported AR inheritance pattern, suggesting the potential presence of a second, possibly deep intronic variant that is difficult to detect. Another patient (HSP-051) exhibited a double heterozygous variant in *SPG11*, consisting of one PV c.6082 C > T [p.(Gln2028*)] and one VUS c.7174_7177dup [p.(Met2393Argfs*15)]. The VUS is a truncation variant located in the last exon of *SPG11*, which likely results in a protein truncated to less than 10% of its normal length, but current evidence is insufficient to classify it as pathogenic. These findings do not exclude a role for *SPG7* and *SPG11* in Korean AR-HSP; their underrepresentation here may reflect recruitment/ascertainment bias toward adult-onset cases and limitations of our diagnostic approach. Larger, prospectively recruited cohorts including pediatric patients, together with expanded genomic methods, are needed to clarify the contribution of *SPG7*, *SPG11*, and other AR causes.

To clarify testing strategy choices, we compared the scope and utility of available approaches. Targeted panels offer validated coverage of known HSP genes with faster turnaround and lower cost, making them practical first‑line tests in clinical settings. Clinical exome or WES expands gene content and may detect variants in genes not included on panels, while WGS offers additional sensitivity for noncoding and structural variants; long‑read and RNA sequencing further improve detection of complex or deep‑intronic changes. We therefore recommend a tiered diagnostic workflow: panel‑based testing as an initial screen, with escalation to WES/WGS or long‑read/RNA analyses for unresolved cases or when phenotype suggests atypical or autosomal‑recessive causes.

Our study has several limitations. First, the cohort was drawn from patients who underwent genetic testing at a single tertiary hospital, which may limit generalizability of the findings to the broader Korean population. Second, although PV or LPV were identified in 18% of cases, most patients remain without a definitive genetic diagnosis, reflecting both genetic heterogeneity of HSP and the limits of our targeted 54-gene panel. As a targeted panel, it is constrained by the limited gene set and has reduced sensitivity for certain variant classes— deep-intronic variants, repeat expansions, and complex structural variants—so broader and complementary genomic approaches (WES/WGS, long-read sequencing, and RNA sequencing) could improve detection and interpretation. Third, the retrospective, clinician-referred nature of the cohort and phenotypic overlap between HSP and other related neurological disorders (e.g., ALD, cerebrotendinous xanthomatosis, Charlevoix-Saguenay-type spastic ataxia) mean that some diagnostic misclassification is possible. Finally, systematic clinical phenotyping was limited by incomplete records and loss to follow-up inherent to the retrospective design. Despite these limitations, this study represents the largest and most comprehensive genetic analysis of HSP in a Korean population to date, providing valuable insights into the mutational spectrum and highlighting the predominance of *SPAST* and *ATL1* in this cohort. These findings lay a critical foundation for future research on the genetic architecture of HSP in Koreans and the development of diagnostic and therapeutic strategies for patients.

In conclusion, this study provides a comprehensive overview of the genetic landscape of HSP in the Korean population, highlighting the predominance of *SPAST* and *ATL1* mutations and the valuable role of targeted NGS panel testing as a first-line diagnostic tool. Despite these advances, many cases remain unsolved, underscoring the need for broader sequencing approaches such as WES or WGS to discover additional genetic factors. Overall, our findings lay the groundwork for improved diagnostics and personalized treatment strategies in HSP.

## Materials and methods

### Study population

The study population comprised 657 unrelated patients whom the treating clinician suspected of HSP and referred for genetic testing. Inclusion criteria were: (1) a clinical presentation indicative of HSP — progressive lower limb spasticity with gait impairment and muscle weakness, consistent with Harding criteria^25^— and (2) no clear alternative non-hereditary cause of spasticity identified prior to genetic testing (e.g., acute stroke or demyelinating disease). All participants underwent either Sanger sequencing or panel-based NGS testing at a single tertiary hospital between January 2010 and December 2024. Panel-based NGS testing has been implemented since August 2017. Medical records and genetic results were retrospectively reviewed. The genetic study and retrospective data analysis were approved by the institutional review board of Samsung Medical Center (IRB No.: SMC 2025-06-073) and adhered to the tenets of the Declaration of Helsinki. Informed consent for genetic testing was obtained from all investigated subjects; for participants under 19 years of age, the informed consent was obtained from their parents or legal guardians. No images, videos, or details that could identify the patients were used in the study.

### Sanger sequencing

Sanger sequencing was used for the analysis of *SPAST* and *ATL1* gene with custom-designed primers (available on request). Genomic DNA was isolated from peripheral blood leukocytes of patient using a Wizard Genomic DNA purification kit (Promega, Madison, WI, USA) according to the manufacturer’s instructions. Sequencing of the targeted region was performed using an ABI 3730 Genetic Analyzer (Applied Biosystems, Foster City, CA, USA). Sequence variants were analyzed using Sequencher software (Gene Codes Co, Ann Arbor, MI, USA) and compared with the reference sequences for *SPAST* (NM_014946.3) and *ATL1* (NM_015915.4).

### Panel-based NGS testing

DNA underwent sequencing with a targeted panel designed to selectively capture 54 genes associated with HSP and related disorders (Table S4). Target enrichment using hybrid-capture method was performed with the TWIST Hybridization kit (Twist Bioscience, South San Francisco, CA, USA) according to the manufacturer’s protocol, including library pooling, hybridization, target capture, and wash. Targeted DNA fragments were sequenced on a NextSeq550 or NovaSeq 6000 platform (Illumina, San Diego, CA, USA). The mean sequencing depth across target regions was 196.1× (range, 151.6–337.5×), with 99.99% of target bases covered at ≥ 30×. These metrics meet our laboratory’s internal quality criteria for reliable detection of germline heterozygous variants. Exon-level coverage is verified for all target genes; any exon failing internal quality control is re-sequenced or confirmed by Sanger, and suspected CNVs are followed up with orthogonal testing (e.g., MLPA). Sequence reads were aligned against human reference genome GRCh37 (hg19) using Burrows-Wheeler Aligner (BWA v0.7.17) and then the output sorted to remove PCR duplicates using Picard v2.19.0. Using GATK workflow (v4.1.2), we processed the data for local indel realignment and base quality recalibration. Variant calling was performed using Strelka2 (v2.9.10), VarDict, and Genome Analysis Toolkit (GATK, v4.1.2), and variants were annotated using SnpEff (v4.3). Data analysis was performed on variants located within ± 25 base pairs of the coding exon, considering population frequency, effect on the encoded protein, and conservation and expression of the variant. Variants were classified in accordance with the 2015 American College of Medical Genetics and Genomics/Association for Molecular Pathology (ACMG/AMP) variant interpretation guidelines^26^. The specifications and strengths of each evidence type were refined based on the general recommendations outlined by the ClinGen Sequence Variant Interpretation working group (https://clinicalgenome.org/tools/clingen-variant-classification-guidance/, last accessed in July 2025). The variant results of tests conducted between 2010 and 2015 were reinterpreted in accordance with the ACMG/AMP guidelines published in 2015 and the subsequent ClinGen guidelines. The variant data analyzed in this study are provided in the Supplementary tables (Tables S1and S5).

### Criteria for genetic diagnosis

Genetic diagnosis was carried out by identifying pathogenic variants (PVs) and likely pathogenic variants (LPVs) according to the established variant interpretation guidelines. The genetic inheritance mode defined in the OMIM database for each gene was used in this process (https://www.omim.org/ last accessed in May 2025). The criteria for genetic diagnosis were as follows: (1) if one PV or LPV was identified in an autosomal dominant HSP (AD-HSP) gene, the variant was deemed to cause AD-HSP; (2) if two PVs or LPVs were identified in an autosomal recessive HSP (AR-HSP) gene, either in a homozygous or compound heterozygous state, the variants were considered causative of AR-HSP; (3) if one PV or LPV was identified in an X-linked HSP gene (XL-HSP) in male patients, the variant was determined to cause XL-HSP; (4) for genes exhibiting both autosomal dominant and recessive inheritance patterns in HSP, such as *ALDH18A1* and *SPG7*, the mode of inheritance for each variant was established based on the published literature; and (5) PV or LPV in genes that are not causative of HSP but present with similar clinical features, such as *ABCD1* and *CYP27A1*, that were identified in accordance with the known mode of inheritance were considered to cause an hereditary neurological disorder other than HSP. Patients whose genetic diagnosis met any of those five criteria were classified as *solved*, and those for whom the causative gene or variant could not be determined were classified as *unsolved*. In addition, the identification of a single PV or LPV in AR gene, or the presence of a PV or LPV in a double heterozygous state alongside a variant of uncertain significance (VUS), was considered inconclusive. Such cases were classified as *unsolved*.

### Additional molecular tests for candidate variant validation

#### Reverse transcription-PCR (RT-PCR) and sequencing for splice site variants

Splicing effects of intronic and synonymous variants detected by sequencing were predicted in silico using SpliceAI^27^, with a delta score > 0.2 considered indicative of a deleterious splicing impact^28^. For candidate variants meeting this threshold, RT-PCR and Sanger sequencing were performed on available patient RNA using primers specifically designed to assess aberrant splicing. Total RNA was extracted from the lymphocyte fraction isolated from peripheral blood using TRIzol methods and reverse transcribed into cDNA using Omniscript^®^ Reverse Transcriptase (QIAGEN, Hilden, Germany). The presence of the aberrant mRNA transcript was confirmed when an abnormal band was identified on agarose gel electrophoresis of the tested sample compared to the control. For the subsequent Sanger validation, PCR products were sequenced on an ABI 3730 Genetic Analyzer (Applied Biosystems) using a BigDye Terminator Cycle sequencing kit (Applied Biosystems). Sequences were analyzed using Sequencher software (Gene Codes Corp.). Gel images adhered to digital image and integrity policies and were obtained from samples processed in parallel from the same experiment.

#### Detection of copy number variations (CNVs)

To screen for CNVs in NGS data, we used a previously published in-house CNV detection method^29^. The coverage depth of each exon in the target region from the NGS data was normalized by dividing it by the mean depth of coverage for the sample. The ratio of normalized coverage of the test sample to the median normalized coverage of control samples sequenced in the same batch was calculated. A copy number gain or loss was defined as when the log2 ratio was greater than 0.4 or less than − 0.55, respectively, with the normal range established between those two values. CNV analysis was performed for all patients who underwent NGS testing and was applied across every gene on our 54-gene panel.

To confirm suspected CNVs identified from the NGS screening, multiplex ligation-dependent probe amplification (MLPA) was performed on patients with available DNA, using an appropriate MLPA probemix according to the manufacturer’s instructions (MRC Holland, Amsterdam, Netherlands). PCR products were separated using an ABI 3500 analyzer (Applied Biosystems), and the copy number analysis was conducted using Coffalyser software (MRC Holland). Peak heights were normalized, and potential deletion or duplication events were identified when the normalized peak ratios were less than 0.75 or greater than 1.30, respectively.

### Statistical analysis

Baseline characteristics are presented as median and range for age and as frequency and percentage for categorical variables. Comparisons between the solved and unsolved study population subsets were conducted using the chi-square test, Fisher’s exact test, or Mann-Whitney U-test, as appropriate. The calculations were performed with VassarStats (http://vassarstats.net/), and 95% confidence intervals were calculated for each value. All *P* values were based on two-sided comparisons, and *P* values < 0.05 were considered statistically significant.

## Supplementary Information

Below is the link to the electronic supplementary material.

Supplementary Material 1

## Data Availability

All variant-level data generated and analyzed during the current study are provided in Supplementary information.
