# Supplementary material for: Genetic landscape of hereditary spastic paraplegias in the Korean population
Source: Sci Rep. 2026 May 30;16:24844. doi: 10.1038/s41598-026-53107-0 (PMC13458517; doi:10.1038/s41598-026-53107-0)
Supplement: Supplementary file 1 [file 41598_2026_53107_MOESM1_ESM.docx]

**Supplementary Information**

**Genetic landscape of hereditary spastic paraplegias in the Korean population**

**Fig. S1.** Diagram of the study design illustrating diagnostic rates by genetic testing methods and genetic spectrum results by mode of inheritance in patients with suspected hereditary spastic paraplegia. ^*^The study includes 15 patients who underwent both Sanger sequencing and NGS. ^†^*ABCD1*, *ALDH18A1*, *CYP27A1*, *KIF5A*, *REEP1*, and *SACS.*

Abbreviations: AD, autosomal dominant; AR, autosomal recessive; HSP, hereditary spastic paraplegia; LPV, likely pathogenic variant; NGS, next generation sequencing; PV, pathogenic variant; XL, X-linked.

**Fig. S2.** Age distribution and causative gene analysis in genetically solved patients with suspected hereditary spastic paraplegia (HSP). (A) Age distribution of entire study cohort and the differences in solved rates by age group. (B) Proportion of causative genes and (C) autosomal dominant (AD), X-linked (XL), and autosomal recessive (AR) inheritance patterns among all genetically solved patients with suspected HSP.

**Fig. S3.** Validation of candidate *SPAST* variants by reverse transcription (RT)-PCR and sequencing. (A) c.1004+3_1004+6del: cDNA fragments approximately 232 bp in size deleting exon 6 was detected in the patient but not in the control. (B) c.1005-3T>G: cDNA fragments approximately 530 bp in size, containing a partial retained intron 6, was detected in the patient but not in the control. (C) c.1712_1728+84del: cDNA fragments approximately 446 bp in size deleting exon 16 was detected in the patient but not in the control (control, 487 bp). (D) Genomic DNA sequence showed soft-clipped sequence and abrupt decreased read depth due to c.1712_1728+84del variant at exon 16 and the flanking intron region of *SPAST*. The RT-PCR images shown are one representative clinical scan per variant; where available, full-length membrane scans are included. Patient identifiers on original membranes have been replaced with study codes.

**Fig. S4.** *SPAST* exon-level deletion/duplications where copy number change was suspected through NGS panel sequencing and verified through MLPA analysis. The upper blue and lower red lines represent the threshold values of normality. Black dots inside between the horizontal lines indicated normal alleles, while red dots under the lower red line indicate a deletion. MLPA analysis revealed deletions in exons 5–7 (A), 8–9 (B), 15–17 (C), and a larger deletion encompassing exons 1–17 (D) of *SPAST*. In addition, a duplication was identified in exon 8 (E).

Abbreviation: Del, deletion; Dup, duplication; MLPA, multiplex ligation-dependent probe amplification.

**Table S1.** Summary of clinical findings and genetic results of 121 genetically solved patients with suspected hereditary spastic paraplegia

| **Patient** | **Sex** | **Age at onset, years** | **Age at genetic testing, years** | **Family history** | **SPG type** | **Transcript** | | **Gene** | | **DNA change** | | **Predicted effect** | | **Sensory problem** | | **Urinary problem** | | **Babinski reflex** | |  |
| --- | --- | --- | --- | --- | --- | --- | --- | --- | --- | --- | --- | --- | --- | --- | --- | --- | --- | --- | --- | --- |
| HSP-026 | F | <10 | 23 | + | Pure | | NM_014946.3 | | *SPAST* | | Del: exon 15-17 | | | | - | | - | | + | |
| HSP-029 | F | <10 | 40 | + | Pure | | NM_015915.4 | | *ATL1* | | c.536C>A | | p.(Ser179Tyr) | | N/A | | - | | + | |
| HSP-037 | F | 50s | 60 | + | Pure | | NM_014946.3 | | *SPAST* | | c.1763C>T | | p.(Ser588Phe) | | + | | + | | + | |
| HSP-038 | F | N/A | 31 | + | Pure | | NM_015915.4 | | *ATL1* | | c.740A>C | | p.(His247Pro) | | - | | - | | + | |
| HSP-040 | M | N/A | 48 | + | N/A | | NM_014946.3 | | *SPAST* | | c.1011del | | p.(Ala338Leufs*7) | | N/A | | N/A | | N/A | |
| HSP-047 | M | 30s | 57 | + | Pure | | NM_014946.3 | | *SPAST* | | c.1712_1728+84del | | p.? | | - | | + | | N/A | |
| HSP-048 | M | 40s | 47 | + | N/A | | NM_000033.3 | | *ABCD1* | | c.1895C>T | | p.(Thr632Ile) | | + | | - | | - | |
| HSP-053 | F | 20s | 43 | + | Pure | | NM_014946.3 | | *SPAST* | | c.571delC | | p.(Arg191Alafs*5) | | N/A | | + | | - | |
| HSP-059 | M | 50s | 56 | N/A | Complicated | | NM_015915.4 | | *ATL1* | | c.35-3C>T | | p.? | | - | | + | | N/A | |
| HSP-060 | M | N/A | 69 | N/A | N/A | | NM_014946.3 | | *SPAST* | | c.1493+2T>G | | p.? | | N/A | | N/A | | N/A | |
| HSP-061 | M | N/A | 24 | N/A | N/A | | NM_014946.3 | | *SPAST* | | c.1413+3_1413+6del | | p.? | | N/A | | N/A | | N/A | |
| HSP-075 | F | N/A | 50 | N/A | N/A | | NM_014946.3 | | *SPAST* | | c.1741C>T | | p.(Arg581*) | | N/A | | N/A | | N/A | |
| HSP-078 | F | N/A | 50 | N/A | N/A | | NM_014946.3 | | *SPAST* | | c.571del | | p.(Arg191Alafs*5) | | N/A | | N/A | | N/A | |
| HSP-085 | M | N/A | 1 | N/A | N/A | | NM_014946.3 | | *SPAST* | | c.1729-1G>C | | p.? | | N/A | | N/A | | N/A | |
| HSP-095 | M | 40s | 50 | N/A | N/A | | NM_014946.3 | | *SPAST* | | c.730_732delinsG | | p.(Asn244Valfs*21) | | N/A | | N/A | | N/A | |
| HSP-097 | M | N/A | 66 | N/A | N/A | | NM_014946.3 | | *SPAST* | | c.1763C>A | | p.(Ser588Tyr) | | N/A | | N/A | | N/A | |
| HSP-098 | F | N/A | 18 | N/A | N/A | | NM_014946.3 | | *SPAST* | | c.1495C>T | | p.(Arg499Cys) | | N/A | | N/A | | N/A | |
| HSP-100 | F | 50s | 57 | + | Pure | | NM_014946.3 | | *SPAST* | | c.1098+1G>A | | p.? | | - | | + | | N/A | |
| HSP-102 | M | N/A | 44 | N/A | N/A | | NM_014946.3 | | *SPAST* | | c.1066G>A | | p.(Glu356Lys) | | N/A | | N/A | | N/A | |
| HSP-111 | F | N/A | 64 | N/A | N/A | | NM_014946.3 | | *SPAST* | | c.1114A>G | | p.(Arg372Gly) | | N/A | | N/A | | N/A | |
| HSP-113 | M | N/A | 51 | N/A | N/A | | NM_014946.3 | | *SPAST* | | c.1154G>T | | p.(Gly385Val) | | N/A | | N/A | | N/A | |
| HSP-120 | M | N/A | 18 | N/A | N/A | | NM_014946.3 | | *SPAST* | | c.1031T>A | | p.(Ile344Lys) | | N/A | | N/A | | N/A | |
| HSP-122 | M | 40s | 53 | + | Pure | | NM_014946.3 | | *SPAST* | | c.911dupC | | p.(Thr305Tyrfs*6) | | - | | - | | - | |
| HSP-138 | M | 60s | 72 | - | Pure | | NM_014946.3 | | *SPAST* | | c.1004+3_1004+6del | | p.(Gly291Trpfs*5) | | - | | - | | N/A | |
| HSP-139 | M | N/A | 62 | N/A | N/A | | NM_014946.3 | | *SPAST* | | c.1245+1G>A | | p.? | | N/A | | N/A | | N/A | |
| HSP-295 | M | N/A | 50 | N/A | N/A | | NM_014946.3 | | *SPAST* | | c.1081C>T | | p.(Pro361Ser) | | N/A | | N/A | | N/A | |
| HSP-148 | F | N/A | 56 | N/A | N/A | | NM_014946.3 | | *SPAST* | | c.391dup | | p.(Leu131Profs*5) | | N/A | | N/A | | N/A | |
| HSP-150 | M | 30s | 39 | + | Pure | | NM_014946.3 | | *SPAST* | | c.67_85dup | | p.(Leu29Glnfs*25) | | - | | - | | + | |
| HSP-151 | M | 30s | 45 | N/A | Pure | | NM_000033.3 | | *ABCD1* | | c.1876G>A | | p.(Ala626Thr) | | N/A | | + | | N/A | |
| HSP-153 | M | N/A | 48 | N/A | N/A | | NM_014946.3 | | *SPAST* | | c.1378C>T | | p.(Arg460Cys) | | N/A | | N/A | | N/A | |
| HSP-156 | M | N/A | 39 | N/A | N/A | | NM_014946.3 | | *SPAST* | | c.1066G>A | | p.(Glu356Lys) | | N/A | | N/A | | N/A | |
| HSP-174 | F | N/A | 41 | N/A | N/A | | NM_014946.3 | | *SPAST* | | c.1137_1142del | | p.(Leu379_Leu380del) | | N/A | | N/A | | N/A | |
| HSP-182 | M | N/A | 50 | N/A | N/A | | NM_014946.3 | | *SPAST* | | c.571del | | p.(Arg191Alafs*5) | | N/A | | N/A | | N/A | |
| HSP-191 | F | N/A | 56 | N/A | N/A | | NM_014946.3 | | *SPAST* | | c.990_993del | | p.(Asn330Lysfs*14) | | N/A | | N/A | | N/A | |
| HSP-194 | M | N/A | 50 | N/A | N/A | | NM_014946.3 | | *SPAST* | | c.1712_1728+84del101 | | p.? | | N/A | | N/A | | N/A | |
| HSP-202 | M | N/A | 32 | N/A | N/A | | NM_014946.3 | | *SPAST* | | c.1039C>T | | p.(Gln347*) | | N/A | | N/A | | N/A | |
| HSP-358 | F | N/A | 79 | N/A | N/A | | NM_014946.3 | | *SPAST* | | c.1081C>T | | p.(Pro361Ser) | | N/A | | N/A | | N/A | |
| HSP-205 | M | N/A | 53 | N/A | N/A | | NM_014946.3 | | *SPAST* | | c.1082C>G | | p.(Pro361Arg) | | N/A | | N/A | | N/A | |
| HSP-208 | F | N/A | 26 | N/A | N/A | | NM_014946.3 | | *SPAST* | | c.1082C>G | | p.(Pro361Arg) | | N/A | | N/A | | N/A | |
| HSP-219 | M | 40s | 57 | + | Pure | | NM_014946.3 | | *SPAST* | | c.1413+4A>G | | p.? | | - | | + | | N/A | |
| HSP-220 | F | N/A | 61 | N/A | N/A | | NM_014946.3 | | *SPAST* | | c.1112T>G | | p.(Leu371Arg) | | N/A | | N/A | | N/A | |
| HSP-226 | M | N/A | 67 | N/A | N/A | | NM_014946.3 | | *SPAST* | | c.1702C>T | | p.(Gln568*) | | N/A | | N/A | | N/A | |
| HSP-235 | M | 40s | 65 | + | Pure | | NM_014946.3 | | *SPAST* | | c.1216A>G | | p.(Ile406Val) | | - | | - | | + | |
| HSP-240 | M | N/A | 46 | N/A | N/A | | NM_014946.3 | | *SPAST* | | c.1537-11A>G | | p.? | | N/A | | N/A | | N/A | |
| HSP-241 | M | N/A | 57 | N/A | N/A | | NM_014946.3 | | *SPAST* | | c.1685G>A | | p.(Arg562Gln) | | N/A | | N/A | | N/A | |
| HSP-246 | M | N/A | 44 | N/A | N/A | | NM_014946.3 | | *SPAST* | | c.1537-11A>G | | p.? | | N/A | | N/A | | N/A | |
| HSP-253 | F | N/A | 57 | N/A | N/A | | NM_014946.3 | | *SPAST* | | c.443G>A | | p.(Trp148*) | | N/A | | N/A | | N/A | |
| HSP-257 | M | N/A | 20 | N/A | N/A | | NM_014946.3 | | *SPAST* | | c.734C>G | | p.(Ser245*) | | N/A | | N/A | | N/A | |
| HSP-264 | F | 40s | 52 | + | N/A | | NM_014946.3 | | *SPAST* | | c.1114A>G | | p.(Arg372Gly) | | N/A | | N/A | | N/A | |
| HSP-269 | F | N/A | 35 | + | N/A | | NM_014946.3 | | *SPAST* | | c.1170G>T | | p.(Met390Ile) | | N/A | | N/A | | N/A | |
| HSP-271 | M | N/A | 42 | N/A | N/A | | NM_014946.3 | | *SPAST* | | c.1098+1G>A | | p.? | | N/A | | N/A | | N/A | |
| HSP-273 | F | 30s | 40 | + | Pure | | NM_015915.4 | | *ATL1* | | c.35-3C>T | | p.? | | N/A | | N/A | | N/A | |
| HSP-274 | M | N/A | 58 | N/A | N/A | | NM_014946.3 | | *SPAST* | | c.1215_1219del | | p.(Asn405Lysfs*36) | | N/A | | N/A | | N/A | |
| HSP-287 | M | N/A | 42 | N/A | N/A | | NM_014946.3 | | *SPAST* | | c.1717_1720del | | p.(Ser573Profs*4) | | N/A | | N/A | | N/A | |
| HSP-292 | F | 30s | 38 | + | Pure | | NM_014946.3 | | *SPAST* | | c.1238C>G | | p.(Ser413*) | | - | | + | | - | |
| HSP-209 | F | N/A | 28 | N/A | N/A | | NM_014946.3 | | *SPAST* | | c.1082C>G | | p.(Pro361Arg) | | N/A | | N/A | | N/A | |
| HSP-299 | F | N/A | 71 | N/A | N/A | | NM_014946.3 | | *SPAST* | | c.1537-11A>G | | p.? | | N/A | | N/A | | N/A | |
| HSP-310 | M | N/A | 44 | N/A | N/A | | NM_014946.3 | | *SPAST* | | c.870G>A | | p.(Lys290=) | | N/A | | N/A | | N/A | |
| HSP-330 | F | 20s | 30 | + | Pure | | NM_014946.3 | | *SPAST* | | Del: exon 1-17 | | | | N/A | | + | | N/A | |
| HSP-331 | M | 30s | 38 | + | Pure | | NM_014946.3 | | *SPAST* | | c.1114A>G | | p.(Arg372Gly) | | - | | - | | - | |
| HSP-339 | F | <10 | 18 | + | Pure | | NM_015915.4 | | *ATL1* | | c.715C>T | | p.(Arg239Cys) | | - | | - | | - | |
| HSP-343 | M | 40s | 62 | + | Pure | | NM_014946.3 | | *SPAST* | | c.1494-2del | | p.? | | + | | + | | + | |
| HSP-350 | M | N/A | 33 | N/A | N/A | | NM_014946.3 | | *SPAST* | | c.1216A>G | | p.(Ile406Val) | | N/A | | N/A | | N/A | |
| HSP-384 | M | N/A | 55 | N/A | N/A | | NM_014946.3 | | *SPAST* | | c.1082C>G | | p.(Pro361Arg) | | N/A | | N/A | | N/A | |
| HSP-372 | M | N/A | 51 | N/A | N/A | | NM_014946.3 | | *SPAST* | | c.1561_1567del | | p.(Leu521Valfs*7) | | N/A | | N/A | | N/A | |
| HSP-374 | F | 50s | 58 | N/A | Pure | | NM_014946.3 | | *SPAST* | | c.1322-1G>C | | p.? | | N/A | | + | | + | |
| HSP-376 | M | 10s | 14 | + | Pure | | NM_022912.2 | | *REEP1* | | c.302delA | | p.(Lys101Argfs*26) | | - | | - | | - | |
| HSP-381 | M | N/A | 59 | N/A | N/A | | NM_014946.3 | | *SPAST* | | c.1216A>G | | p.(Ile406Val) | | N/A | | N/A | | N/A | |
| HSP-383 | M | N/A | 36 | N/A | N/A | | NM_014946.3 | | *SPAST* | | c.467del | | p.(Leu156Argfs*5) | | N/A | | N/A | | N/A | |
| HSP-404 | F | N/A | 60 | N/A | N/A | | NM_014946.3 | | *SPAST* | | c.1082C>G | | p.(Pro361Arg) | | N/A | | N/A | | N/A | |
| HSP-387 | F | N/A | 36 | + | N/A | | NM_014946.3 | | *SPAST* | | c.1098+1G>A | | p.? | | N/A | | N/A | | N/A | |
| HSP-392 | F | N/A | 59 | N/A | N/A | | NM_014946.3 | | *SPAST* | | c.1511dup | | p.(Tyr505Ilefs*7) | | N/A | | N/A | | N/A | |
| HSP-396 | M | <10 | 7 | + | Pure | | NM_022912.2 | | *REEP1* | | Del: exon 6-7 | |  | | - | | + | | N/A | |
| HSP-400 | M | 30s | 44 | - | Pure | | NM_014946.3 | | *SPAST* | | c.1005-3T>G | | p.? | | - | | - | | + | |
| HSP-401 | F | N/A | 41 | N/A | N/A | | NM_014946.3 | | *SPAST* | | c.1157A>G | | p.(Asn386Ser) | | N/A | | N/A | | N/A | |
| HSP-140 | F | N/A | 42 | N/A | N/A | | NM_014946.3 | | *SPAST* | | c.1082C>T | | p.(Pro361Leu) | | N/A | | N/A | | N/A | |
| HSP-405 | M | 40s | 43 | + | Pure | | NM_014946.3 | | *SPAST* | | c.1114A>G | | p.(Arg372Gly) | | N/A | | N/A | | N/A | |
| HSP-424 | F | 10s | 39 | + | Pure | | NM_014946.3 | | *SPAST* | | c.1240A>G | | p.(Lys414Glu) | | - | | + | | N/A | |
| HSP-425 | M | N/A | 1 | N/A | N/A | | NM_014946.3 | | *SPAST* | | c.1606C>T | | p.(Gln536*) | | N/A | | N/A | | N/A | |
| HSP-432 | F | 30s | 42 | + | Pure | | NM_014946.3 | | *SPAST* | | c.1712_1728+84del | | p.(Glu563Aspfs*8) | | N/A | | - | | + | |
| HSP-435 | M | N/A | 58 | + | N/A | | NM_014946.3 | | *SPAST* | | c.1127dupG | | p.(Gly377Argfs*17) | | N/A | | N/A | | N/A | |
| HSP-437 | M | N/A | 49 | N/A | N/A | | NM_014946.3 | | *SPAST* | | c.302C>A | | p.(Ser101*) | | N/A | | N/A | | N/A | |
| HSP-450 | F | N/A | 49 | N/A | N/A | | NM_014946.3 | | *SPAST* | | c.1098+1G>A | | p.? | | N/A | | N/A | | N/A | |
| HSP-453 | M | 50s | 61 | + | N/A | | NM_000033.3 | | *ABCD1* | | c.112C>T | | p.(Gln38*) | | - | | - | | N/A | |
| HSP-457 | F | N/A | 55 | N/A | N/A | | NM_014946.3 | | *SPAST* | | c.1072_1075delinsTC | | p.(Val358Serfs*8) | | N/A | | N/A | | N/A | |
| HSP-464 | M | N/A | 56 | N/A | N/A | | NM_014946.3 | | *SPAST* | | c.1245+1G>A | | p.? | | N/A | | N/A | | N/A | |
| HSP-482 | F | 50s | 60 | - | N/A | | NM_000033.3 | | *ABCD1* | | c.1628C>T | | p.(Pro543Leu) | | + | | - | | N/A | |
| HSP-488 | F | N/A | 51 | N/A | N/A | | NM_014946.3 | | *SPAST* | | c.1245+1G>A | | p.? | | N/A | | N/A | | N/A | |
| HSP-497 | F | N/A | 23 | N/A | N/A | | NM_014946.3 | | *SPAST* | | c.571del | | p.(Arg191Alafs*5) | | N/A | | N/A | | N/A | |
| HSP-504 | F | 50s | 59 | - | N/A | | NM_000784.3 | | *CYP27A1* | | c.1001T>C  c.1420C>T | | p.(Met334Lys)  p.(Arg474Trp) | | N/A | | N/A | | N/A | |
| HSP-506 | M | 10s | 25 | + | Pure | | NM_014946.3 | | *SPAST* | | c.753del | | p.(Val252Leufs*2) | | N/A | | + | | - | |
| HSP-507 | M | N/A | 45 | N/A | N/A | | NM_014946.3 | | *SPAST* | | c.1685G>A | | p.(Arg562Gln) | | N/A | | N/A | | N/A | |
| HSP-516 | M | 10s | 34 | + | Pure | | NM_022912.2 | | *REEP1* | | c.2T>C | | p.(Met1?) | | - | | - | | N/A | |
| HSP-519 | M | 10s | 35 | + | Pure | | NM_014946.3 | | *SPAST* | | c.1240A>G | | p.(Lys414Glu) | | + | | - | | - | |
| HSP-521 | M | 50s | 61 | + | Pure | | NM_014946.3 | | *SPAST* | | c.1215_1219del | | p.(Asn405Lysfs*36) | | N/A | | + | | - | |
| HSP-523 | M | 10s | 25 | + | N/A | | NM_014363.5 | | *SACS* | | c.2903_2906del  c.13217del | | p.(Asp968Valfs*13)  p.(Thr4406Argfs*46) | | + | | - | | N/A | |
| HSP-533 | F | 50s | 71 | + | Pure | | NM_014946.3 | | *SPAST* | | Del: exon 8-9 | |  | | N/A | | - | | N/A | |
| HSP-537 | M | N/A | 36 | N/A | N/A | | NM_014946.3 | | *SPAST* | | c.302C>A | | p.(Ser101*) | | N/A | | N/A | | N/A | |
| HSP-539 | M | N/A | 45 | N/A | N/A | | NM_014946.3 | | *SPAST* | | c.1537-1G>A | | p.? | | N/A | | N/A | | N/A | |
| HSP-547 | F | N/A | 58 | N/A | N/A | | NM_014946.3 | | *SPAST* | | c.376_377dup | | p.(Ile127Thrfs*35) | | N/A | | N/A | | N/A | |
| HSP-549 | M | N/A | 23 | N/A | N/A | | NM_014946.3 | | *SPAST* | | c.1435A>T | | p.(Arg479*) | | N/A | | N/A | | N/A | |
| HSP-551 | M | N/A | 26 | N/A | N/A | | NM_015915.4 | | *ATL1* | | c.1193C>T | | p.(Ser398Phe) | | N/A | | N/A | | N/A | |
| HSP-564 | F | 40s | 57 | N/A | Pure | | NM_014946.3 | | *SPAST* | | c.583C>G | | p.(Leu195Val) | | N/A | | N/A | | N/A | |
| HSP-571 | M | 40s | 48 | N/A | N/A | | NM_000033.3 | | *ABCD1* | | c.250C>T | | p.(Pro84Ser) | | - | | N/A | | N/A | |
| HSP-579 | M | N/A | 40 | N/A | N/A | | NM_014946.3 | | *SPAST* | | c.870+1del | | p.? | | N/A | | N/A | | N/A | |
| HSP-580 | F | 50s | 66 | - | Pure | | NM_014946.3 | | *SPAST* | | Del: exon 5-7 | | | | N/A | | + | | N/A | |
| HSP-587 | F | 50s | 60 | + | Pure | | NM_014946.3 | | *SPAST* | | c.577C>T | | p.(Gln193*) | | - | | + | | N/A | |
| HSP-593 | F | N/A | 53 | N/A | N/A | | NM_014946.3 | | *SPAST* | | c.571del | | p.(Arg191Alafs*5) | | N/A | | N/A | | N/A | |
| HSP-599 | M | <10 | 13 | + | Pure | | NM_014946.3 | | *SPAST* | | c.577C>T | | p.(Gln193*) | | N/A | | - | | N/A | |
| HSP-602 | F | N/A | 55 | N/A | N/A | | NM_014946.3 | | *SPAST* | | c.1378C>T | | p.(Arg460Cys) | | N/A | | N/A | | N/A | |
| HSP-608 | F | 30s | 43 | - | Pure | | NM_014946.3 | | *SPAST* | | c.519_520del | | p.(Arg173Serfs*2) | | N/A | | - | | N/A | |
| HSP-610 | M | N/A | 65 | N/A | N/A | | NM_014946.3 | | *SPAST* | | c.1712_1728+84del | | p.? | | N/A | | N/A | | N/A | |
| HSP-611 | F | 50s | 58 | + | Pure | | NM_014946.3 | | *SPAST* | | Dup: exon 8 | |  | | N/A | | - | | N/A | |
| HSP-618 | M | <10 | 34 | + | Pure | | NM_022912.2 | | *REEP1* | | c.303+1G>T | | p.? | | N/A | | + | | N/A | |
| HSP-621 | M | 30s | 44 | + | Pure | | NM_014946.3 | | *SPAST* | | c.1751A>T | | p.(Asp584Val) | | - | | - | | + | |
| HSP-628 | M | 10s | 20 | + | Pure | | NM_015915.4 | | *ATL1* | | c.1483C>T | | p.(Arg495Trp) | | N/A | | N/A | | N/A | |
| HSP-639 | F | N/A | 69 | N/A | N/A | | NM_014946.3 | | *SPAST* | | c.571del | | p.(Arg191Alafs*5) | | N/A | | N/A | | N/A | |
| HSP-643 | M | 40s | 45 | - | Pure | | NM_014946.3 | | *SPAST* | | c.1218_1219del | | p.(Ile406Metfs*36) | | - | | - | | - | |
| HSP-647 | F | 20s | 25 | - | Pure | | NM_002860.3 | | *ALDH18A1* | | c.755G>A | | p.(Arg252Gln) | | - | | - | | N/A | |
| HSP-648 | M | N/A | 63 | N/A | N/A | | NM_014946.3 | | *SPAST* | | c.1413+3_1413+6del | | p.? | | N/A | | N/A | | N/A | |
| HSP-651 | M | 40s | 42 | - | Pure | | NM_004984.2 | | *KIF5A* | | c.838C>T | | p.(Arg280Cys) | | - | | - | | N/A | |

Abbreviations: Del, deletion; F, female; M, male; N/A, not available; SPG, spastic paraplegia.

**Table S2.** Summary of clinical findings and genetic results of seven patients with hereditary spastic paraplegia-related disorders

| **Patient** | **Sex** | **Age at onset, years** | **Age at genetic testing, years** | **Family history** | **SPG type** | **Transcript** | **Gene** | **Nucleotide change** | **Predicted effect** | **Sensory problem** | **Urinary problem** | **Babinski reflex** | **Laboratory test** |
| --- | --- | --- | --- | --- | --- | --- | --- | --- | --- | --- | --- | --- | --- |
| HSP-048 | M | 40s | 47 | + | N/A | NM_000033.3 | *ABCD1* | c.1895C>T | p.Thr632Ile | + | - | - | N/A |
| HSP-151 | M | 30s | 45 | N/A | Pure | NM_000033.3 | *ABCD1* | c.1876G>A | p.Ala626Thr | N/A | + | N/A | N/A |
| HSP-453 | M | 50s | 61 | + | N/A | NM_000033.3 | *ABCD1* | c.112C>T | p.Gln38* | - | - | N/A | ^*^C26:0 — 3.86 µmol/L (elevated); C26:0/C22:0 ratio — 0.091 (elevated) |
| HSP-482 | F | 50s | 60 | - | N/A | NM_000033.3 | *ABCD1* | c.1628C>T | p.Pro543Leu | + | - | N/A | ^*^C26:0 — 2.13 µmol/L (elevated); C26:0/C22:0 ratio — 0.052 (elevated) |
| HSP-571 | M | 40s | 48 | N/A | N/A | NM_000033.3 | *ABCD1* | c.250C>T | p.Pro84Ser | - | N/A | N/A | ^*^C26:0 — 1.91 µmol/L (elevated); C26:0/C22:0 ratio — 0.03 (elevated) |
| HSP-504 | F | 50s | 59 | - | N/A | NM_000784.3 | *CYP27A1* | c.1001T>C  c.1420C>T | p.Met334Lys  p.Arg474Trp | N/A | N/A | N/A | N/A |
| HSP-523 | M | 10s | 25 | + | N/A | NM_014363.5 | *SACS* | c.2903_2906del  c.13217del | p.Asp968Valfs*13  p.Thr4406Argfs*46 | + | - | N/A | N/A |

Abbreviations: Del, deletion; F, female; M, male; N/A, not available; SPG, spastic paraplegia.

^*^Reference ranges were C26:0 0–1.3 µmol/L and C26:0/C22:0 ratio 0–0.023; measurements were performed by gas chromatography–mass spectrometry.

**Table S3.** Inconclusive cases as a heterozygous carrier or compound heterozygote of an autosomal recesssive HSP gene

| **Patient** | **Sex** | **Age at onset, years** | **Age at genetic testing, years** | **Family history** | **Gene** | **Transcript** | **Nucleotide change** | **Predicted effect** | **Zygosity** | **Class** | **ACMG criteria** | **Remarks** |
| --- | --- | --- | --- | --- | --- | --- | --- | --- | --- | --- | --- | --- |
| HSP-259 | M | N/A | 25 | + | *SPG7* | NM_003119.2 | c.244C>T | p.(Gln82*) | Het | PV | PVS1, PM2, PM3_P | Previously reported as homozygous in a case of late-onset HSP (PMID 30747022) |
| HSP-051 | F | 30s | 58 | + | *SPG11* | NM_025137.3 | c.6082C>T  c.7174_7177dup | p.(Gln2028*)  p.(Met2393Argfs*15) | Het  Het | PV  VUS | PVS1, PM3, PM2  PVS1_M, PM2 | HSP complicated by dementia, severe cognitive impairment, and abnormal hearing |

Abbreviations: ACMG, American College of Medical Genetics and Genomics; F, female; Het, heterozygous; HSP, hereditary spastic paraplegia; M, male; LPV, likely pathogenic variant; PV, pathogenic variant; VUS, variant of uncertain significance.

**Table S4**. Genes included in the targeted panel sequencing: associated disorders, inheritance, and supporting evidence for inclusion

| **Gene** | **HGNC** | **Inheritance** | **Phenotype** | **Evidence** | **East Asian Genetic Publications (PMID)** |
| --- | --- | --- | --- | --- | --- |
| *ABCD1* | HGNC:61 | XL | Adrenoleukodystrophy, OMIM:300100 | PanelApp expert review, Green; literature | 31777199, 34649108, 34291142 |
| *ALDH18A1* | HGNC:9722 | AD/AR | Spastic paraplegia 9A, autosomal dominant, OMIM:601162; Spastic paraplegia 9B, autosomal recessive, OMIM:616586 | PanelApp expert review, Green; literature | 26297558, 29915212, 31289639 |
| *ALS2* | HGNC:443 | AR | Amyotrophic lateral sclerosis 2, juvenile, OMIM:205100;Primary lateral sclerosis, juvenile, OMIM:606353;Spastic paralysis, infantile onset ascending, OMIM:607225 | PanelApp expert review, Green; literature | 25433428 |
| *AP4B1* | HGNC:572 | AR | Spastic paraplegia 47, autosomal recessive, OMIM:614066 | PanelApp expert review, Green; literature | 30337681, 31525725, 36122674 |
| *AP4E1* | HGNC:573 | AR | Spastic paraplegia 51, autosomal recessive, OMIM:613744 | PanelApp expert review, Green; literature | 30337681 |
| *AP4M1* | HGNC:574 | AR | Spastic paraplegia 50, autosomal recessive, OMIM:612936 | PanelApp expert review, Green; literature | 30337681, 31525725 |
| *AP4S1* | HGNC:575 | AR | Spastic paraplegia 52, autosomal recessive, OMIM:614067 | PanelApp expert review, Green; literature | 30337681 |
| *AP5Z1* | HGNC:22197 | AR | Spastic paraplegia 48, autosomal recessive, OMIM:613647 | PanelApp Expert Review Red: literature | 31289639 |
| *ATL1* | HGNC:11231 | AD | Spastic paraplegia 3A, autosomal dominant, OMIM:182600 | PanelApp expert review, Green; literature | 20947813, 26208798, 31236401 |
| *ARSA* | HGNC:713 | AR | Metachromatic leukodystrophy, OMIM:250100 | PanelApp expert review, Green; literature | 32617873 |
| *B4GALNT1* | HGNC:4117 | AR | Spastic paraplegia 26, autosomal recessive, OMIM:609195 | PanelApp expert review, Green; literature | 33638609 |
| *BSCL2* | HGNC:15832 | AD | Silver spastic paraplegia syndrome, OMIM:270685 | PanelApp expert review, Green; literature | 25487175 |
| *C12orf65* | HGNC:26784 | AR | Spastic paraplegia 55, autosomal recessive, OMIM:615035 | PanelApp expert review, Green; literature | 31753091 |
| *CAPN1* | HGNC:1476 | AR | Spastic paraplegia 76 autosomal recessive, OMIM: 616907 | PanelApp expert review, Green; literature | 31023339 |
| *CYP27A1* | HGNC:2605 | AR | Cerebrotendinous xanthomatosis, OMIM:213700 | PanelApp expert review, Green; literature | 36803198 |
| *CYP2U1* | HGNC:20582 | AR | Spastic paraplegia 56, autosomal recessive, OMIM:615030 | PanelApp expert review, Green; literature | 23176821, 24337409, 27679996 |
| *CYP7B1* | HGNC:2652 | AR | Spastic paraplegia 5A, autosomal recessive , OMIM:270800 | PanelApp expert review, Green; literature | 33771085 |
| *DDHD1* | HGNC:19714 | AR | Spastic paraplegia 28, autosomal recessive, OMIM: 609340 | PanelApp expert review, Green; literature | 23176821 |
| *DDHD2* | HGNC:29106 | AR | Spastic paraplegia 54, autosomal recessive, OMIM: 615033 | PanelApp expert review, Green; literature | 24337409, 27679996 |
| *ENTPD1* | HGNC:3363 | AR | Spastic paraplegia 64, autosomal recessive, OMIM:615683 | PanelApp expert review, Green; literature | 33771085 |
| *ERLIN1* | HGNC:16947 | AR | Hereditary spastic paraplegia;Spastic paraplegia 62, OMIM:615681 | PanelApp expert review, Green; literature | 41251124 |
| *ERLIN2* | HGNC:1356 | AD/AR | Spastic paraplegia 18, autosomal recessive, OMIM: 611225 | PanelApp expert review, Green; literature | 40225166 |
| *FA2H* | HGNC:21197 | AR | Spastic paraplegia 35, autosomal recessive, OMIM:612319 | PanelApp expert review, Green; literature | 24359114 |
| *GAD1* | HGNC:4092 | AR | Cerebralpalsy,spasticquadriplegic,1,OMIM: 603513 | PanelApp Expert Review Red: literature | 33634263 |
| *GALC* | HGNC:4115 | AR | Krabbe disease, OMIM:245200 | PanelApp Expert Review Red: literature | 31185936 |
| *GBA2* | HGNC:18986 | AR | Spastic paraplegia 46, autosomal recessive, OMIM: 614409 | PanelApp expert review, Green; literature | 23332916, 24337409, 33569519 |
| *GJC2* | HGNC:17494 | AR | Spastic paraplegia 44, autosomal recessive, OMIM: 613206 | PanelApp Expert Review Red: literature | 27057822 |
| *HSPD1* | HGNC:5261 | AD/AR | Leukodystrophy, hypomyelinating, 4, OMIM:612233 (AR);Spastic paraplegia 13, autosomal dominant, OMIM:605280 (AD) | PanelApp expert review, Green; literature | 41431411 |
| *KIDINS220* | HGNC:29508 | AD | Spastic paraplegia, intellectual disability, nystagmus, and obesity OMIM:617296 | PanelApp expert review, Green; literature | 39109120 |
| *KIF1A* | HGNC:888 | AD/AR | Spastic paraplegia 30, autosomal dominant, OMIM:610357;Spastic paraplegia 30, autosomal recessive, OMIM:610357 | PanelApp expert review, Green; literature | 36155026, 36972935 |
| *KIF1C* | HGNC:6317 | AR | Spastic ataxia 2, autosomal recessive, OMIM:611302 | PanelApp expert review, Green; literature | 41236093 |
| *KIF5A* | HGNC:6323 | AD | Spastic paraplegia 10, autosomal dominant, OMIM:604187 | PanelApp expert review, Green; literature | 32888732 |
| *L1CAM* | HGNC:6470 | XL | CRASH syndrome, OMIM:303350;MASA syndrome, OMIM:303350;Hydrocephalus due to aqueductal stenosis, OMIM:307000;Hydrocephalus with congential idiopathic intestinal pseudoobstruction, OMIM:307000;Hydrocephalus with Hirschsprung disease, OMIM:307000 | PanelApp expert review, Green; literature | 22554690 |
| *NIPA1* | HGNC:17043 | AD | Spastic paraplegia 6, autosomal dominant, OMIM: 600363 | PanelApp expert review, Green; literature | 17928003 |
| *NT5C2* | HGNC:8022 | AR | Spastic paraplegia 45, autosomal recessive, 613162 | PanelApp expert review, Green; literature | 28884889 |
| *PLP1* | HGNC:9086 | XL | Spastic paraplegia 2, X-linked, OMIM:312920 | PanelApp expert review, Green; literature | 22554690, 30337681 |
| *PNPLA6* | HGNC:16268 | AR | Spastic paraplegia 39, autosomal recessive, OMIM:612020 | PanelApp expert review, Green; literature | 33141049 |
| *REEP1* | HGNC:25786 | AD | Spastic paraplegia 31, autosomal dominant, OMIM: 610250 | PanelApp expert review, Green; literature | 25045380 |
| *REEP2* | HGNC:17975 | AD/AR | ?Spastic paraplegia 72, autosomal recessive, OMIM: 615625; Spastic paraplegia 72, autosomal dominant, OMIM:615625 | PanelApp expert review, Green; literature | 34715294 |
| *RTN2* | HGNC:10468 | AD/AR | Spastic paraplegia 12, autosomal dominant, OMIM:604805 | PanelApp expert review, Green; literature | 35684947 |
| *SACS* | HGNC:10519 | AR | Spastic ataxia, Charlevoix-Saguenay type, OMIM: 270550 | PanelApp expert review, Green; literature | 38928084 |
| *SLC12A6* | HGNC:10914 | AD/AR | Charcot-Marie-Tooth disease, axonal, type 2II, OMIM:620068;Agenesis of the corpus callosum with peripheral neuropathy, OMIM:218000 | PanelApp expert review, Green; literature | 22554690 |
| *SLC16A2* | HGNC:10923 | XL | Allan-Herndon-Dudley syndrome, OMIM:300523 | PanelApp expert review, Green; literature | 39029020 |
| *SOD1* | HGNC:11179 | AR | Spastic tetraplegia and axial hypotonia, progressive, OMIM:618598 | PanelApp expert review, Green; literature | 12480087, 21964799 |
| *SPART* | HGNC:18514 | AR | Troyer syndrome, OMIM:275900;Spastic paraplegia 20 | PanelApp expert review, Green; literature | 31535723 |
| *SPAST* | HGNC:11233 | AD | Spastic paraplegia 4, autosomal dominant, OMIM:182601 | PanelApp expert review, Green; literature | 26208798, 25045380, 32493220 |
| *SPG11* | HGNC:11226 | AR | Spastic paraplegia 11, autosomal recessive, OMIM: 604360 | PanelApp expert review, Green; literature | 18835492, 27018819, 28933964 |
| *SPG21* | HGNC:20373 | AR | Spastic Paraplegia, Recessive, OMIM: 248900 | PanelApp expert review, Green; literature | 24492745 |
| *SPG7* | HGNC:11237 | AD/AR | Spastic paraplegia 7, autosomal recessive, OMIM:607259 | PanelApp expert review, Green; literature | 30497413, 39894496 |
| *TECPR2* | HGNC:19957 | AR | Spastic paraplegia 49, autosomal recessive, OMIM: 615031 | PanelApp Expert Review Red: literature | 35130874 |
| *TFG* | HGNC:11758 | AR | Spastic paraplegia 57, autosomal recessive, OMIM:615658 | PanelApp expert review, Green; literature | 37890998 |
| *WASHC5* | HGNC:28984 | AD | Spastic paraplegia 8, autosomal dominant, OMIM:603563 | PanelApp expert review, Green; literature | 38028608 |
| *ZFYVE26* | HGNC:20761 | AR | Spastic paraplegia 15, autosomal recessive, OMIM:270700 | PanelApp expert review, Green; literature | 33771085, 33637369, 37681008 |
| *ZFYVE27* | HGNC:26559 | AD | Spastic paraplegia 33, autosomal dominant | PanelApp Expert Review Red: literature | 36573383, 29980238 |

Abbreviations: AD, autososmal dominant; AR, autosomal recessive; PMID, PubMed ID; XL, X-linked.

Panelapp: <https://panelapp.genomicsengland.co.uk>

**Table S5**. Variants of uncertain significance identified patients with suspected hereditary spastic paraplegia of this study

| **Gene** | **Transcript** | **Nucleotide change** | **Predicted effect** | **Variant type** | **REVEL** | **dbSNP** | **AF (Global)** | **AF (Korean)** |
| --- | --- | --- | --- | --- | --- | --- | --- | --- |
| *ABCD1* | NM_000033 | c.359G>T | p.(Arg120Leu) | missense |  |  | . | . |
| *ABCD1* | NM_000033 | c.559G>T | p.(Asp187Tyr) | missense |  |  |  |  |
| *ABCD1* | NM_000033 | c.656T>A | p.(Leu219His) | missense |  |  |  |  |
| *ABCD1* | NM_000033 | c.809C>T | p.(Ala270Val) | missense |  |  |  |  |
| *ABCD1* | NM_000033 | c.1093G>C | p.(Val365Leu) | missense |  |  |  |  |
| *ABCD1* | NM_000033 | c.1144A>C | p.(Thr382Pro) | missense | 0.856 |  |  |  |
| *ABCD1* | NM_000033 | c.1585G>A | p.(Gly529Ser) | missense |  |  |  |  |
| *ABCD1* | NM_000033 | c.1651G>T | p.(Gly551Cys) | missense |  |  |  |  |
| *ALDH18A1* | NM_002860 | c.905G>A | p.(Arg302Lys) | missense |  |  |  |  |
| *ALDH18A1* | NM_002860 | c.1315G>A | p.(Gly439Ser) | missense | 0.5 | rs141292408 | 4.38E-05 | 0 |
| *ALS2* | NM_020919 | c.1565C>T | p.(Pro522Leu) | missense | 0.5 | rs760588436 | 1.60E-05 |  |
| *AP4B1* | NM_006594 | c.1894G>C | p.(Glu632Gln) | missense | 0.2 | rs186623784 | 2.79E-05 |  |
| *AP4E1* | NM_007347 | c.1396A>T | p.(Ile466Phe) | missense |  | rs751064747 | | 0.01 |
| *AP4E1* | NM_007347 | c.2731A>G | p.(Thr911Ala) | missense |  | rs747533607 | 3.19E-05 | 0 |
| *AP4M1* | NM_004722 | c.1187C>T | p.(Ser396Leu) | missense |  | rs772203028 | | 0 |
| *ATL1* | NM_015915 | c.686C>G | p.(Ala229Gly) | missense |  |  | 8E-06 |  |
| *ATL1* | NM_015915 | c.1005C>G | p.(Ile335Met) | missense |  |  |  |  |
| *ATL1* | NM_015915 | c.1612T>A | p.(Tyr538Asn) | missense | 0.3 | rs200185276 | 3.59E-05 | 0 |
| *ARSA* | NM_000487 | c.511G>A | p.(Asp171Asn) | missense |  | rs74315466 | 0.0002 | 0 |
| *ARSA* | NM_000487 | c.514G>A | p.(Gly172Ser) | missense |  | rs74315271 | |  |
| *ARSA* | NM_000487 | c.982G>A | p.(Val328Met) | missense | 0.8 | rs143994992 | 0.0005 | 0.01 |
| *ARSA* | NM_000487 | c.1297C>G | p.(Leu433Val) | missense |  | rs201693608 | | 0 |
| *ARSA* | NM_000487 | c.1345G>A | p.(Gly449Arg) | missense | 0.5 | rs117341984 | 0.0003 | 0 |
| *BSCL2* | NM_001122955 | c.986G>A | p.(Arg329Gln) | missense | 0.2 | rs771322168 | 1.59E-05 | 0 |
| *BSCL2* | NM_001122955 | c.1300T>C | p.(Ser434Pro) | missense | 0.2 | rs199584887 | 1.59E-05 |  |
| *BSCL2* | NM_001122955 | c.1309G>C | p.(Ala437Pro) | missense | 0.3 | rs199787351 | 2.39E-05 | 0 |
| *C12orf65* | NM_152269 | c.242G>A | p.(Cys81Tyr) | missense |  |  |  |  |
| *C12orf65* | NM_152269 | c.307C>G | p.(Gln103Glu) | missense | 0.1 | rs777447851 | 1.61E-05 | 0 |
| *C12orf65* | NM_152269 | c.445C>G | p.(Leu149Val) | missense | 0.1 |  |  |  |
| *CAPN1* | NM_001198868 | c.517G>A | p.(Gly173Arg) | missense | 0.4 | rs199993600 | 0.0004 |  |
| *CAPN1* | NM_001198868 | c.1846C>T | p.(Arg616Cys) | missense | 0.3 | rs139570056 | 6.84E-05 | 0 |
| *CYP27A1* | NM_000784 | c.316G>A | p.(Val106Met) | missense | 0.629 | rs374769409 | 0 | 0.0005 |
| *CYP27A1* | NM_000784 | c.355C>T | p.(Arg119Trp) | missense | 0.768 | rs369294392 | 0.0002 |  |
| *CYP27A1* | NM_000784 | c.368A>C | p.(Lys123Thr) | missense | 0.491 | rs778424069 | 1.591E-5 | 0.0005 |
| *CYP27A1* | NM_000784 | c.401A>C | p.(Lys134Thr) | missense | 0.214 |  | 3.98E-6 |  |
| *CYP27A1* | NM_000784 | c.545T>C | p.(Ile182Thr) | missense | 0.246 | rs556745324 | 8.748E-5 | 0.0014 |
| *CYP27A1* | NM_000784 | c.563G>T | p.(Arg188Leu) | missense |  |  |  |  |
| *CYP27A1* | NM_000784 | c.674G>A | p.(Arg225His) | missense | 0.863 | rs753275244 | 1.989E-5 |  |
| *CYP27A1* | NM_000784 | c.694T>C | p.(Ser232Pro) | missense |  |  |  |  |
| *CYP27A1* | NM_000784 | c.784C>T | p.(Arg262Cys) | missense | 0.43 | rs778371330 | 4.374E-5 | 0.0009 |
| *CYP27A1* | NM_000784 | c.809G>A | p.(Arg270Gln) | missense |  | rs556715266 | 4.374E-5 |  |
| *CYP27A1* | NM_000784 | c.1001T>A | p.(Met334Lys) | missense |  |  |  |  |
| *CYP27A1* | NM_000784 | c.1028C>T | p.(Thr343Met) | missense | 0.876 | rs372194079 | 0.0001 |  |
| *CYP27A1* | NM_000784 | c.1033A>C | p.(Thr345Pro) | Missense |  |  |  |  |
| *CYP2U1* | NM_183075 | c.1004G>C | p.(Ser335Thr) | missense | 0.2 | rs142944337 | 0.0001 | 0.01 |
| *CYP2U1* | NM_183075 | c.1300T>C | p.(Tyr434His) | missense | 0.6 | rs755199255 | 8.95E-06 |  |
| *CYP7B1* | NM_004820 | c.177C>A | p.(Val59Val) | missense | . | rs763202430 | 8.35E-05 | 0 |
| *CYP7B1* | NM_004820 | c.530C>T | p.(Thr177Met) | missense | 0.3 | rs145152682 | 0.0004 |  |
| *CYP7B1* | NM_004820 | c.806A>G | p.(Asp269Gly) | missense |  | rs753799233 | 3.59E-05 | 0 |
| *DDHD1* | NM_001160147 | c.700G>A | p.(Ala234Thr) | missense |  | rs200233648 | 3.59E-05 | 0 |
| *DDHD1* | NM_001160147 | c.1588G>A | p.(Asp530Asn) | missense |  |  |  |  |
| *DDHD2* | NM_015214 | c.139T>G | p.(Cys47Gly) | missense |  | rs200883537 | 0.0002 | 0.01 |
| *DDHD2* | NM_015214 | c.1130C>T | p.(Ser377Leu) | missense |  | rs145943165 | | 0 |
| *ERLIN2* | NM_007175 | c.464A>G | p.(Asp155Gly) | missense | 1 |  |  |  |
| *FA2H* | NM_024306 | c.316A>T | p.(Thr106Ser) | missense | 0.2 |  |  |  |
| *FA2H* | NM_024306 | c.794T>G | p.(Phe265Cys) | missense | 0.8 | rs149133141 | 6.85E-05 | 0 |
| *FA2H* | NM_024306 | c.794T>G | p.(Phe265Cys) | missense |  |  |  |  |
| *GAD1* | NM_000817 | c.80C>A | p.(Thr27Lys) | missense |  | rs77655188 | | 0.01 |
| *GAD1* | NM_000817 | c.184C>T | p.(Arg62Cys) | missense | 0.3 | rs548422399 | 9.55E-05 | 0 |
| *GAD1* | NM_000817 | c.395T>C | p.(Phe132Ser) | missense |  | rs376982509 | 4.37E-05 | . |
| *GALC* | NM_000153 | c.56C>T | p.(Ala19Val) | missense | 0.27 | . |  |  |
| *GALC* | NM_000153 | c.175G>A | p.(Gly59Ser) | missense | 0.963 | rs764757647 | 0 |  |
| *GALC* | NM_000153 | c.208C>A | p.(Leu70Ile) | missense |  |  |  |  |
| *GALC* | NM_000153 | c.890T>G | p.(Ile297Ser) | missense |  |  |  |  |
| *GALC* | NM_000153 | c.1400C>T | p.(Thr467Ile) | missense |  |  |  |  |
| *GALC* | NM_000153 | c.1415G>T | p.(Gly472Val) | missense |  |  |  |  |
| *GALC* | NM_000153 | c.1566C>A | p.(Asp522Glu) | missense |  | rs758827171 |  | . |
| *GALC* | NM_000153 | c.1976C>T | p.(Pro659Leu) | missense |  |  |  | . |
| *GJC2* | NM_020435 | c.1066C>A | p.(Gln356Lys) | missense | 0.4 | rs146841026 | 9.43E-05 |  |
| *GJC2* | NM_020435 | c.1199C>A | p.(Ala400Glu) | missense | 0.3 | rs761261049 | 0.0003 | 0 |
| *KIDINS220* | NM_020738 | c.1462G>A | p.(Gly488Arg) | missense | 0.4 | rs770692330 | 2.22E-05 |  |
| *KIDINS220* | NM_020738 | c.2701C>T | p.(Arg901Trp) | missense | 0.5 | rs372110208 | 8.02E-06 |  |
| *KIDINS220* | NM_020738 | c.3460G>A | p.(Gly1154Ser) | missense | 0.1 | rs374088731 | 2.50E-05 | 0 |
| *KIDINS220* | NM_020738 | c.4961C>G | p.(Ser1654Cys) | missense | 0.3 | rs778969597 | 4.41E-05 | 0 |
| *KIF1A* | NM_004321 | c.877A>T | p.(Asn293Tyr) | missense |  | rs1042391791 | 6.297E-6 |  |
| *KIF1A* | NM_004321 | c.1630C>T | p.(Arg544Trp) | missense | 0.585 | rs369141291 | 1.222E-5 |  |
| *KIF1A* | NM_004321 | c.1835C>T | p.(Thr612Met) | missense |  | rs760338913 |  |  |
| *KIF1A* | NM_004321 | c.3251C>T | p.(Pro1084Leu) | missense | 0.598 |  | 4.015E-6 |  |
| *KIF1A* | NM_004321 | c.3400G>A | p.(Asp1134Asn) | missense | 0.688 | rs200257048 | 0.0001 | 0.0005 |
| *KIF1A* | NM_004321 | c.3568C>T | p.(Arg1190Cys) | missense | 0.418 | rs199998986 | 0.0002 | 0.0027 |
| *KIF1A* | NM_004321 | c.4241C>G | p.(Pro1414Arg) | missense | 0.204 | . | 1.774E-5 |  |
| *KIF1A* | NM_004321 | c.4624G>A | p.(Asp1542Asn) | missense | 0.053 | rs200141437 | 0.0004 | 0.0005 |
| *KIF1A* | NM_004321 | c.4652G>A | p.(Arg1551Gln) | missense |  | rs376658420 | 0.0002 | . |
| *KIF1C* | NM_006612 | c.899A>T | p.(Tyr300Phe) | missense | 0.8 | rs760721111 | 3.58E-05 |  |
| *KIF5A* | NM_004984 | c.2120G>A | p.(Arg707Gln) | missense | 0.5 |  | 3.99E-06 |  |
| *KIF5A* | NM_004984 | c.2557G>A | p.(Asp853Asn) | missense | 0.5 | rs780457980 | 1.99E-05 | 0 |
| *PNPLA6* | NM_006702 | c.335G>A | p.(Arg112Gln) | missense | 0.1 | rs376396266 | 1.09E-05 |  |
| *PNPLA6* | NM_006702 | c.901C>G | p.(Leu301Val) | missense | 0 |  |  |  |
| *SACS* | NM_014363 | c.755G>T | p.(Gly252Val) | missense | 0.652 |  |  |  |
| *SACS* | NM_014363 | c.877C>A | p.(Leu293Ile) | missense | 0.416 |  |  |  |
| *SACS* | NM_014363 | c.1066A>G | p.(Ile356Val) | missense | 0.17 | rs148286091 | 5.171E-5 | 0.0027 |
| *SACS* | NM_014363 | c.1186A>G | p.(Asn396Asp) | missense | 0.564 | rs78629788 | 3.978E-6 |  |
| *SACS* | NM_014363 | c.2494T>C | p.(Ser832Pro) | missense | 0.26 | rs549957998 | 0.0002 | 0.0027 |
| *SACS* | NM_014363 | c.2643G>C | p.(Glu881Asp) | missense | 0.184 | rs200517685 | 0.0003 | 0.0032 |
| *SACS* | NM_014363 | c.2996T>C | p.(Ile999Thr) | missense | 0.824 | rs371869943 | 9.587E-5 | 0.0005 |
| *SACS* | NM_014363 | c.3193T>C | p.(Phe1065Leu) | missense | 0.557 | rs770947700 | 8.676E-6 | 0.0005 |
| *SACS* | NM_014363 | c.3752T>C | p.(Ile1251Thr) | missense | 0.584 | rs76872266 | 0.0006 |  |
| *SACS* | NM_014363 | c.3970A>G | p.(Ile1324Val) | missense | 0.269 | rs775801970 | 7.962E-6 |  |
| *SACS* | NM_014363 | c.5528T>A | p.(Leu1843Gln) | missense |  |  |  |  |
| *SACS* | NM_014363 | c.5608G>A | p.(Val1870Met) | missense | 0.493 | rs752750902 | 7.978E-6 |  |
| *SACS* | NM_014363 | c.6150A>G | p.(Thr2050Thr) | missense |  |  | 4.015e-06 |  |
| *SACS* | NM_014363 | c.6226T>G | p.(Leu2076Val) | missense | 0.651 | rs763841642 | 8.009E-6 | 0.0009 |
| *SACS* | NM_014363 | c.6278G>T | p.(Arg2093Leu) | missense | 0.51 |  |  |  |
| *SACS* | NM_014363 | c.6422A>G | p.(Asn2141Ser) | missense |  |  | 4.004E-6 | 0.0014 |
| *SACS* | NM_014363 | c.6778A>G | p.(Ile2260Val) | missense | 0.328 | rs202110208 | 2.789E-5 | 0.0009 |
| *SACS* | NM_014363 | c.7223A>G | p.(Asp2408Gly) | missense | 0.233 | rs146509108 | 2.794E-5 | 0.0009 |
| *SACS* | NM_014363 | c.8441A>G | p.(Asn2814Ser) | missense | 0.267 |  |  |  |
| *SACS* | NM_014363 | c.8482A>G | p.(Ser2828Gly) | missense | 0.242 | rs9552930 | 0.0003 | 0.0036 |
| *SACS* | NM_014363 | c.8579A>G | p.(Asn2860Ser) | missense | 0.355 | rs201294520 | 0.0001 | 0.0014 |
| *SACS* | NM_014363 | c.9283C>G | p.(Pro3095Ala) | missense | 0.788 | rs201899988 | 5.584E-5 | 0.0023 |
| *SACS* | NM_014363 | c.9299C>A | p.(Ser3100Tyr) | missense | 0.242 | rs150615169 | 0.0006 | 0.0068 |
| *SACS* | NM_014363 | c.9661G>A | p.(Val3221Met) | missense | 0.428 | rs142867964 | 0.0001 | 0.0073 |
| *SACS* | NM_014363 | c.9805G>A | p.(Asp3269Asn) | missense | 0.755 | rs754144089 | 1.196E-5 |  |
| *SACS* | NM_014363 | c.10157C>A | p.(Ala3386Glu) | Missense |  |  |  |  |
| *SACS* | NM_014363 | c.10909A>G | p.(Met3637Val) | missense | 0.169 | rs150309559 | 0.0004 | 0.005 |
| *SACS* | NM_014363 | c.10982C>T | p.(Ala3661Val) | missense | 0.329 | rs36061856 | 0.001 |  |
| *SACS* | NM_014363 | c.11048A>G | p.(Asn3683Ser) | missense | 0.262 | rs138609508 | 9.578E-5 | 0.0005 |
| *SACS* | NM_014363 | c.11725C>A | p.(Leu3909Ile) | missense | 0.435 |  |  |  |
| *SACS* | NM_014363 | c.12670A>G | p.(Ile4224Val) | missense | 0.194 |  |  |  |
| *SACS* | NM_014363 | c.13039A>G | p.(Ile4347Val) | missense | 0.177 | rs200939906 | 9.984E-5 | 0.0032 |
| *SLC12A6* | NM_133647 | c.964G>A | p.(Gly322Ser) | missense | 0.9 | rs75235010 | 0.0002 | 0.01 |
| *SLC12A6* | NM_133647 | c.1012C>T | p.(Arg338Cys) | missense | 0.8 | rs77122016 | 0.0004 | 0.01 |
| *SLC12A6* | NM_133647 | c.3013C>T | p.(Arg1005Trp) | missense | 0.7 | rs190611250 | 5.57E-05 | 0 |
| *SOD1* | NM_000454 | c.158A>G | p.(Asp53Gly) | missense | 1 |  |  |  |
| *SOD1* | NM_000454 | c.337A>G | p.(Ile113Val) | Missense |  |  |  |  |
| *SPART* | NM_015087 | c.137A>G | p.(Tyr46Cys) | missense | 0.9 |  |  |  |
| *SPART* | NM_015087 | c.1853C>T | p.(Thr618Ile) | missense | 0.3 | rs772826042 | 4.38E-05 | 0 |
| *SPAST* | NM_014946 | c.616A>G | p.(Lys206Glu) | Missense |  | rs781149761 | 0.000007956 |  |
| *SPAST* | NM_014946 | c.620C>T | p.(Ser207Leu) | missense |  |  | 0.0000082 | 0.0008 |
| *SPAST* | NM_014946 | c.847G>A | p.(Gly283Ser) | missense |  |  |  |  |
| *SPAST* | NM_014946 | c.1112T>G | p.(Leu371Arg) | missense |  |  |  |  |
| *SPAST* | NM_014946 | c.1141T>G | p.(Phe381Val) | Missense |  |  |  |  |
| *SPAST* | NM_014946 | c.1453G>C | p.(Ala485Pro) | missense | 0.965 |  |  |  |
| *SPAST* | NM_014946 | c.1573C>A | p.(Gln525Lys) | missense | 0.566 |  |  |  |
| *SPAST* | NM_014946 | c.1728G>C | p.(Glu576Asp) | missense |  |  |  |  |
| *SPG11* | NM_025137 | c.398G>A | p.(Cys133Tyr) | missense | 0.242 |  |  |  |
| *SPG11* | NM_025137 | c.491C>T | p.(Ser164Leu) | missense | 0.264 | rs148175530 | 0.0004 | 0.0009 |
| *SPG11* | NM_025137 | c.581C>T | p.(Pro194Leu) | missense | 0.089 | rs573482671 | 7.556E-5 | 0.0023 |
| *SPG11* | NM_025137 | c.3067G>A | p.(Glu1023Lys) | missense |  |  |  |  |
| *SPG11* | NM_025137 | c.3320G>C | p.(Cys1107Ser) | missense | 0.098 | rs201271196 | 0.0002 | 0.0014 |
| *SPG11* | NM_025137 | c.3749T>C | p.(Ile1250Thr) | missense | 0.2 | rs763244237 | 8.754E-5 |  |
| *SPG11* | NM_025137 | c.4204C>T | p.(His1402Tyr) | missense | 0.886 | rs756973532 | 1.194E-5 | 0.0014 |
| *SPG11* | NM_025137 | c.4517T>C | p.(Ile1506Thr) | missense |  |  |  |  |
| *SPG11* | NM_025137 | c.4888G>C | p.(Glu1630Gln) | missense | 0.142 | rs368276916 | 3.979E-6 | 0.0009 |
| *SPG11* | NM_025137 | c.5471G>A | p.(Arg1824Gln) | Missense |  |  |  |  |
| *SPG11* | NM_025137 | c.5566G>C | p.(Glu1856Gln) | missense | 0.233 | rs749792714 | 3.978E-6 |  |
| *SPG11* | NM_025137 | c.6224A>G | p.(Asn2075Ser) | missense | 0.085 | rs140824939 | 0.003 |  |
| *SPG11* | NM_025137 | c.6284T>C | p.(Leu2095Ser) | missense | 0.495 | rs185665930 | 0.0007 | 0.0018 |
| *SPG11* | NM_025137 | c.6691A>T | p.(Ile2231Phe) | missense | 0.515 |  |  |  |
| *SPG7* | NM_003119 | c.265G>T | p.(Val89Phe) | missense | 0.412 |  |  |  |
| *SPG7* | NM_003119 | c.543G>C | p.(Gln181His) | missense |  |  |  | 0.0005 |
| *SPG7* | NM_003119 | c.656T>C | p.(Ile219Thr) | missense | 0.547 | rs114255772 | 0.0003 | 0.0055 |
| *SPG7* | NM_003119 | c.1686C>G | p.(Ile562Met) | missense |  | rs201229581 | 7.963E-6 | 0.0005 |
| *SPG7* | NM_003119 | c.2219A>G | p.Tyr740Cys | Missense |  |  |  |  |
| *SPG7* | NM_003119 | c.2342C>G | p.(Thr781Ser) | missense |  |  |  |  |
| *TECPR2* | NM_014844 | c.1033G>A | p.(Asp345Asn) | missense | 0.249 |  |  |  |
| *TECPR2* | NM_014844 | c.1606G>C | p.(Gly536Arg) | missense | 0.064 | rs186595127 | 0.0002 | 0.0014 |
| *TECPR2* | NM_014844 | c.1784C>T | p.(Thr595Met) | missense | 0.058 | rs150893437 | 0.0001 | 0.0005 |
| *TECPR2* | NM_014844 | c.1792G>A | p.(Gly598Arg) | missense | 0.041 | rs772792595 | 0.0001 | 0.0023 |
| *TECPR2* | NM_014844 | c.3467C>T | p.(Ala1156Val) | missense |  | rs762126011 | 8.055e-06 | 0.0009 |
| *TECPR2* | NM_014844 | c.3521A>G | p.(Tyr1174Cys) | missense |  | rs767911807 | 8.129E-5 | 0.0005 |
| *TECPR2* | NM_014844 | c.3867C>A | p.(Asn1289Lys) | missense |  |  |  |  |
| *TECPR2* | NM_014844 | c.3980G>A | p.(Arg1327His) | missense | 0.127 | rs764421543 | 1.691E-5 |  |
| *TECPR2* | NM_014844 | c.4116G>T | p.(Pro1372Pro) | missense |  | rs777406369 | 0.0001 | 0.0032 |
| *TFG* | NM_006070 | c.524A>G | p.(Asp175Gly) | missense | 0.2 |  |  |  |
| *TFG* | NM_006070 | c.981A>G | p.(Gln327Gln) | missense |  | rs759578675 | 1.19E-05 | 0 |
| *TFG* | NM_006070 | c.1148G>A | p.(Arg383His) | missense | 0.3 | rs146202800 | 2.39E-05 | 0 |
| *WASHC5* | NM_014846 | c.779T>C | p.(Val260Ala) | missense | 0.9 | rs747008037 | 3.98E-06 |  |
| *ZFYVE26* | NM_015346 | c.809G>A | p.(Gly270Asp) | missense | 0 | rs753619935 | |  |
| *ZFYVE26* | NM_015346 | c.4066T>G | p.(Cys1356Gly) | missense | 0.2 | rs149276487 | 0.002 | 0.01 |
| *ZFYVE26* | NM_015346 | c.4388A>G | p.(Gln1463Arg) | missense |  |  |  |  |
| *ZFYVE26* | NM_015346 | c.5225C>G | p.(Ser1742Cys) | missense | 0.2 | rs193244014 | 0.0003 | 0.01 |

Abbreviations: AF, allele frequency. Global allele frequencies are based on gnomAD v2 (exome). Koreran allele frequencies are from Korean Reference Genome Database (KRGDB, PMID: 32133509).
